# Synthesis and Evaluation of 1-(1-(Benzo[*b*]thiophen-2-yl)cyclohexyl)piperidine (BTCP) Analogues as Inhibitors of Trypanothione Reductase

**DOI:** 10.1002/cmdc.200900098

**Published:** 2009-06-25

**Authors:** Stephen Patterson, Deuan C Jones, Emma J Shanks, Julie A Frearson, Ian H Gilbert, Paul G Wyatt, Alan H Fairlamb

**Affiliations:** a Division of Biological Chemistry & Drug Discovery, College of Life Sciences, University of DundeeDundee, DD1 5EH (UK), Fax: (+44) 1382 385542

**Keywords:** BTCP, inhibitors, medicinal chemistry, *trypanosoma brucei*, trypanothione reductase

## Abstract

Thirty two analogues of phencyclidine were synthesised and tested as inhibitors of trypanothione reductase (TryR), a potential drug target in trypanosome and leishmania parasites. The lead compound BTCP (**1**, 1-(1-benzo[*b*]thiophen-2-yl-cyclohexyl) piperidine) was found to be a competitive inhibitor of the enzyme (*K*_i_=1 μm) and biologically active against bloodstream *T. brucei* (EC_50_=10 μm), but with poor selectivity against mammalian MRC5 cells (EC_50_=29 μm). Analogues with improved enzymatic and biological activity were obtained. The structure–activity relationships of this novel series are discussed.

## Introduction

Parasites of the order Kinetoplastida are the causative agents of a number of human and animal diseases including Human African Trypanosomiasis (HAT) (caused by *Trypanosoma brucei rhodesiense and T. b. gambiense*), Chagas’ disease (*T. cruzi*) and the leishmaniases (*Leishmania* sp.). Collectively these diseases have a large unmet disease burden,^1^ with the current therapeutics used to treat them possessing severe limitations.^2^ All of these trypanosomatid parasites use a trypanothione-based redox metabolism,^3^ which is absent in humans. The enzymes of this redox pathway are therefore considered to be attractive targets for the development of new antitrypanosomatid drugs.^4^

One component of the trypanothione-based redox pathway is trypanothione reductase (TryR), which is responsible for reducing trypanothione disulfide to the dithiol trypanothione and in doing so provides reducing equivalents to protect the parasites from oxidative damage.^3^ In *T. brucei* it has been demonstrated that TryR activity is required for parasites to grow in culture and to be infective in a mouse disease model.^5^ Therefore, TryR is a validated drug target, and there are a number of recent reports outlining the discovery and development of inhibitors of this key enzyme.^6^

A recently reported high-throughput screening (HTS) of known bioactive compounds against *T. cruzi* TryR identified a number of novel TryR inhibitors^7^ including the arylcyclohexylamine BTCP^8^ (**1**, 1-(1-benzo[*b*]thiophen-2-yl-cyclohexyl)-piperidine). BTCP (**1**) is an analogue of the anaesthetic drug PCP (**2**, 1-(1-phenyl-cyclohexyl)-piperidine, phenylcyclidine). However, despite the structural similarity between compounds **1** and **2**, they have been shown to possess a different pharmacological selectivity.^8^ BTCP (**1**) is a more potent dopamine uptake inhibitor and has a much lower affinity for the PCP receptor.

BTCP (**1**) was considered to be a promising screening hit for further development due to its low molecular weight (299), low micromolar potency against *T. cruzi* TryR (IC_50_=3.7 μm), a promising ligand efficiency (0.35 kcal mol^−1^ L), lack of activity against the human homologue of TryR, glutathione reductase (GR), and the fact that phencyclidines are known to cross the blood–brain barrier, an essential property for the successful treatment of stage 2 HAT. BTCP (**1**) also has the advantage of being a druglike molecule, in contrast to some of the more potent reported TryR inhibitors, many of which are polyamine analogues6a,d,f designed to mimic the spermidine moiety of the enzyme substrate trypanothione. In addition, there are a number of publications relating to BTCP (**1**) and other phencyclidines detailing both synthetic strategies for analogue synthesis and their associated pharmacological activities.^9^


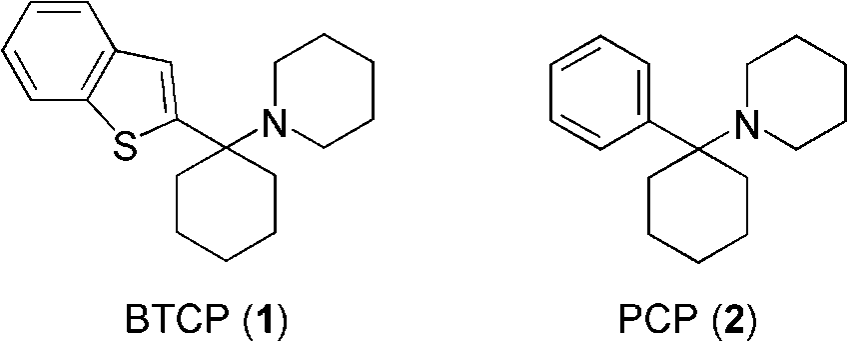


Due to the limitations of the current treatments for HAT, there is a need for the identification of new compound classes displaying antitrypanosomal activity. Therefore, a systematic structure–activity relationship (SAR) analysis of BTCP (**1**) was undertaken to optimise activity against both TryR and the intact parasite *T. brucei*. The results of these investigations are reported herein.

## Results and Discussion

### Biological characterisation of BTCP

In order to determine the validity of BTCP (**1**) as a starting point for a target-driven approach towards the identification of a lead compound for the treatment of HAT, the inhibitory activity of BTCP against *T. brucei* TryR had to be determined. BTCP (**1**) was assayed against *T. brucei* TryR using a HTS format based on a published nonenzymatically coupled assay^10^ and found to have an IC_50_ value of 3.3 μm, confirming its suitability for further investigation. There is no significant difference between the IC_50_ values for **1** against *T. cruzi* (IC_50_=3.7 μm) and *T. brucei* TryR (IC_50_=3.3 μm), which is as expected given the high degree of sequence identity between TryR in the two species (83 % at the amino acid level). A more detailed kinetic analysis established that BTCP is a linear competitive inhibitor of TryR (with respect to trypanothione), with a *K*_i_ value of 1.00±0.08 μm, in good agreement with the IC_50_ value determined in the HTS-format TryR assay.

BTCP (**1**) was assayed against bloodstream form *T. brucei brucei* cells in a HTS-assay format and found to have an EC_50_ value of 10 μm, in close agreement with the previously published EC_50_ value of 14 μm.^7^ BTCP (**1**) was screened against MRC-5 cells in the same 96-well format as for the trypanosome assay giving an EC_50_ value of 29 μm. Unfortunately, the threefold selectivity between MRC-5 and *T. brucei* is suboptimal, but the selectivity is sufficient to warrant further development of the compound series.

### Synthesis of BTCP analogues

There are insufficient commercially available analogues of BTCP (**1**) to establish a comprehensive SAR. Therefore, a chemical synthesis programme was required to support the development of the hit compound. Initial synthetic studies focussed on preparing a diverse collection of BTCP analogues systematically modifying the benzo[*b*]thiophene group, the piperidine ring and the cyclohexyl ring (Table 1). In particular we were interested in carrying out the following modifications to probe for new interactions with the protein: changing the benzo[*b*]thiophene to other aromatic rings, both monocyclic and bicyclic; modifying the size of the piperidine ring and putting heteroatoms into the ring; modifying the size of the cyclohexyl ring and adding substituents to it.

**Table 1:** Analogues of BTCP (**1**) and their inhibitory activities against *T. brucei* TryR and in cell-based assays. See Scheme 1 for the structure of analogues **1**–**19** and Scheme 2 for **23**–**25**.

Compd	Ar	X	Y	*n*^1^	*n*^2^	TryR IC_50_ [μm]	*T. brucei* EC_50_ [μm]
**1** (BTCP)	2-Benzo[*b*]thiophene	CH_2_	CH_2_	1	1	3.3^[a]^	10^[b]^
**2** (PCP)	Benzene	CH_2_	CH_2_	1	1	57	ND
**3**	2-Thiophene	CH_2_	CH_2_	1	1	>100	ND
**4**	4-Phenyl-benzene	CH_2_	CH_2_	1	1	>100	ND
**5**	2-Benzo[*b*]furan	CH_2_	CH_2_	1	1	4.4^[c]^	18
**6**	1-Naphthylene	CH_2_	CH_2_	1	1	>100	ND
**7**	2-Naphthylene	CH_2_	CH_2_	1	1	28	ND
**8**	2-(1-Methylindole)	CH_2_	CH_2_	1	1	36	ND
**9**	2-Benzo[*b*]thiazole	CH_2_	CH_2_	1	1	>100	ND
**10**	3-Benzo[*b*]thiophene	CH_2_	CH_2_	1	1	60	ND
**11**	2-(3-Bromobenzo[*b*]thiophene)	CH_2_	CH_2_	1	1	16	ND
**12**	2-(5-Bromobenzo[*b*]thiophene)	CH_2_	CH_2_	1	1	>100	ND
**13**	2-Benzo[*b*]thiophene	CH_2_	CH_2_	0	1	0.91^[d]^	5.0
**14**	2-Benzo[*b*]thiophene	–	CH_2_	1	1	5.0	13^[e]^
**15**	2-Benzo[*b*]thiophene	O	CH_2_	1	1	11	37
**16**	2-Benzo[*b*]thiophene	NCH_3_	CH_2_	1	1	10	2.1^[f]^
**17**	2-Benzo[*b*]thiophene	CH_2_	CH_2_	1	0	11	35
**18**	2-Benzo[*b*]thiophene	CH_2_	NCH_3_	1	1	0.93	∼15^[g]^
**19**	2-Benzo[*b*]thiophene	CH_2_	n/a	1	n/a	15	27
**23**	n/a	CH_2_	CH_2_	1	1	>100	ND
**24**	n/a	CH_2_	CH_2_	1	1	>100	ND
**25**	n/a	CH_2_	CH_2_	1	1	>100	ND

[a] TryR *K*_i_ 1.00 μm. [b] MRC-5 EC_50_ 29 μm. [c] TryR *K*_i_ 1.46 μm. [d] TryR *K*_i_ 0.26 μm. [e] MRC-5 EC_50_ 22 μm. [f] MRC-5 EC_50_>50 μm. [g] MRC-5 EC_50_>15 μm. ND=not determined. n/a not applicable, structures shown in full.

**Scheme 1:**
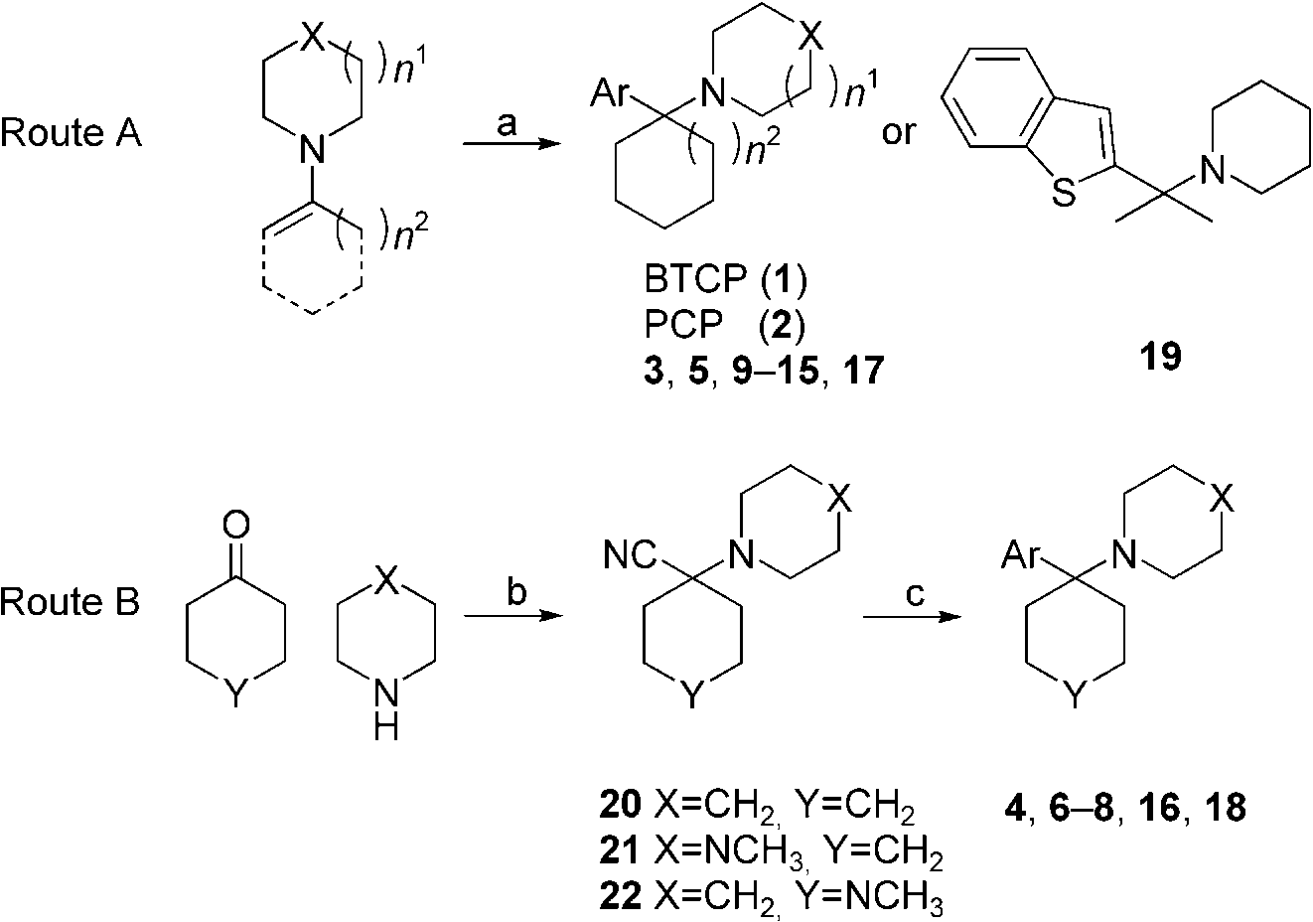
Routes to BTCP analogues **2**–**19**.^11^, ^14^, ^15^ See Table 1 for details of analogue structures. *Reagents and conditions*: a) 1. 1*H*-benzotriazole, Et_2_O, 25 °C, 1 h; 2. ArLi, Et_2_O, 0→25 °C, 16 h; b) acetone cyanohydrin, DMF, MgSO_4_, 50 °C, 2–4 d; c) ArMgBr, Et_2_O, 35 °C, 16 h.

Two different synthetic methodologies were employed to prepare the initial collection: first, addition of aryl lithiums to the benzotriazole adducts of enamines^11^ (Scheme 1, route A); and second, the reaction of aryl Grignards with α-amino nitriles (the Bruylants reaction,^12^ Scheme 1, route B). Route A was successfully employed in reactions where the aryl group was an unsubstituted monocyclic aromatic (**2** & **3**), or when the aryl group was a 5/6 fused bicyclic aromatic (e.g. benzo[*b*]thiophene, compounds **10**, **13**–**15** & **17**). The only exception to the latter observation was that when 1-methylindole was employed in the reaction only a trace amount of the target molecule **8** was formed. Similarly, attempts to prepare naphthyl-substituted phencyclidines (**6** & **7**) via route A were unsuccessful. Preparation of PCP (**2**) from phenyllithium also proceeded in poor yield, suggesting that the Route A methodology is not suited to the synthesis of analogues where a substituted benzene ring is directly attached to the piperidylcyclohexyl moiety. This observation may explain why when 5-bromobenzo[*b*]thiophene was employed as the substrate for lithiation the exclusive product of the reaction was the bromine-substituted BTCP analogue **12**, possibly due to the failure of the generated benzo[*b*]thien-5-yl-lithium species, but not the 5-bromobenzo[*b*]thien-2-yl-lithium species, to react. In contrast, both analogues **10** and **11** were isolated when 3-bromobenzo[*b*]thiophene was employed, due to reactive species formed by lithiation at the 2 position in addition to lithium halogen exchange at the 3 position. The enamine building blocks required for the route A synthesis were obtained from commercial sources, or readily prepared using published methodologies.^13^

Analogues **4**, **6** and **7** have previously been prepared via the Bruylants reaction (route B), therefore, they were prepared following this procedure.^14^ Attempts to prepare the 3-phenyl-benzene isomer of **4** using this methodology were unsuccessful. The indole-containing analogue **8** was also prepared using this procedure. Route B has previously been utilised for the preparation of the amine-containing analogue **16**,^15^ therefore, this route was chosen in preference to route A (Scheme 1). Additionally, the amine-containing analogue **18** was prepared using the Bruylants reaction as the requisite α-amino nitrile **22** was considered to be more synthetically accessible than the substituted enamine that would be required to use route A (Scheme 1).

**Scheme 2:**
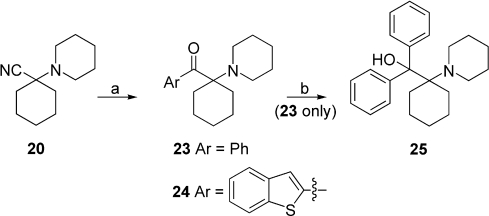
Route to BTCP analogues containing a one carbon “spacer” between the piperidylcyclohexyl and aryl moieties.^16^ *Reagents and conditions*: a) 1. ArLi, Et_2_O, −78→0 °C, 5–16 h, 2. aq HCl, 0 °C, 30 min; b) PhLi, Et_2_O, 0→25 °C, 2.5 h.

In addition, analogues containing a carbonyl “spacer” between the cyclohexylpiperidine core and the aromatic functionality were prepared by reaction of aryl lithiums with alpha-amino nitrile **20** (Scheme 2).^16^ Further reaction of **23** with phenyllithium gave an analogue containing two aryl groups (**25**).

### Trypanothione reductase assay of BTCP analogues

Analogues **2**–**25** were tested for their ability to inhibit *T. brucei* TryR (Table 1) using the HTS assay format previously employed to assay BTCP (**1**). None of the aryl analogues (compounds **2**–**12**) showed an improvement in potency over the hit compound **1**. Analogues where the benzo[*b*]thiophene was replaced with a monocyclic aromatic (compounds **2**–**4**) showed a dramatic reduction in potency against TryR (IC_50_ values 57 to >100 μm), suggesting a requirement for a fused bicyclic aromatic moiety for optimal inhibitor binding. The inhibition values from analogues containing alternative fused bicyclic systems (compounds **5**–**10**) suggest that there is a very specific requirement for a 2-benzo[*b*]thiophene substitution, as demonstrated by testing close isosteres such as 2-naphthyl (compound **7**, IC_50_=28 μm vs 3.3 μm) and analogues containing minor changes in inhibitor structure for example, compound **9** where the benzo[*b*]thiophene is replaced with a benzo[*b*]thiazole (IC_50_>100 μm). Indeed, with the exception of replacing 2-benzo[*b*]thiophene with 2-benzo[*b*]furan (compound **5**) all of the aromatic analogues of BTCP (**1**) were at least one order of magnitude less potent against *T. brucei* TryR (IC_50_ values 28 to >100 μm). The screening results for analogues **11** and **12** demonstrate that it is not possible to substitute 2-benzo[*b*]thiophene at the 5 position, but that substitution at the 3-position gives analogues that retain some activity, albeit reduced. Given these results no further exploration of the aromatic moiety was conducted and all subsequent analogues would incorporate the 2-benzo[*b*]thiophene functionality.

**Figure 1:**
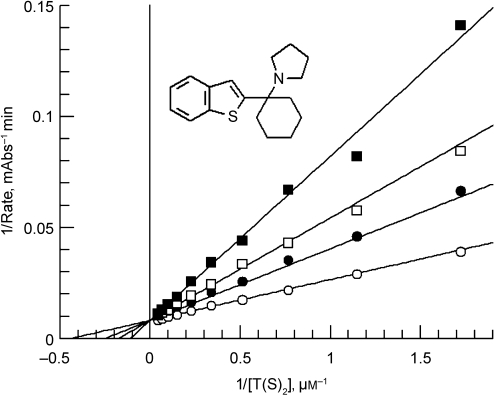
Kinetic analysis of inhibition of *T. brucei* TryR by analogue (**13**). Global fit of data to linear competitive inhibition model presented as a Lineweaver–Burke transformation. Inhibitor concentrations: 0, ○; 0.19 μm, •; 0.39 μm, □; 0.77 μm, ▪.

Analogues **13**–**16** were prepared to investigate the effect of changing the piperidine ring of BTCP (**1**). Exchanging the piperidine for a morpholine or piperazine ring (compounds **15** & **16**) results in a threefold reduction in potency (Table 1), possibly due to the attenuated basicity of the nitrogen atom, or due to the introduction of an additional polar atom (or a combination of both). The acyclic diethylamino analogue (**14**) is of approximately equal potency to the hit compound **1** (IC_50_=5.0 μm vs 3.3 μm). Unfortunately, attempts to prepare more highly substituted acyclic analogues of **1** using route B (Scheme 1) proved unsuccessful. The pyrrolidine-containing analogue **13** was marginally more potent than the hit compound (**1**) (IC_50_=0.91 μm vs 3.3 μm). A full kinetic analysis of analogue **13** showed it to be a linear competitive inhibitor with respect to trypanothione (*K*_i_=0.26±0.01 μm vs 1 μm for BTCP), confirming this mode of inhibition within the BTCP compound series (Figure 1). However, this fourfold increase in potency did not warrant any additional investigation into replacing the piperidine moiety.

The investigation of BTCP cyclohexyl-analogues was limited by synthetic considerations, with just three analogues (**17**–**19**) being prepared. Altering the cyclohexyl moiety by either ring contraction to a cyclopentane ring (**17**), or by replacement with a gem dimethyl substitution (**19**) gave analogues that were three or fivefold less potent, respectively. This suggests that the cyclohexane ring contributes to inhibitory activity by either hydrophobic interactions, or by controlling the orientation by which the other moieties are presented to the protein. The amine-containing analogue **18** showed a slight improvement in potency (IC_50_=0.93 μm vs 3.3 μm) suggesting that it may be possible to introduce a substituted nitrogen at the 4-position of the cyclohexane moiety. Additionally, it may be possible to substitute a carbon atom at the 4 position.

The “spacer”-containing analogues **23**–**25** were all found to be inactive in the *T. brucei* TryR assay (IC_50_>100 μm). Therefore, direct attachment of the aromatic moiety to the cyclohexylpiperidine core is probably an absolute requirement for TryR inhibition within this series. The inactivity of these analogues combined with the failure to significantly increase potency by substitution of the aromatic, or piperidine moieties, meant that substitution at the 4-position of the cyclohexyl ring became the only focus of further investigations (see below).

### Cell-based assays of BTCP analogues

A subset of the analogues prepared as part of the initial diverse BTCP analogue collection (compounds **1**, **5** & **13**–**19**) were assayed for their ability to inhibit the growth of *T. brucei* in culture (Table 1). With the exception of compound **16**, the analogues displayed a decrease in potency between the enzyme and cellular assays of between 2- and 15-fold. Although it is not possible to draw a reliable correlation with this small subset, this level of decrease and its consistency between analogues suggests that inhibition of TryR could be the cause of the inhibition of parasite growth and that it is not the result of an off-target effect.

Additional analogues (**14**, **16** & **18**) were subjected to the MRC-5 counter screen and their selectivity between MRC-5 cells and *T. brucei* was found to be ∼1- to >20-fold. Although this low selectivity is disadvantageous, it may increase in analogues with improved inhibitory activity against TryR.

### Synthesis and TryR assay of BTCP analogues substituted at the 4-position of the cyclohexyl ring

**Scheme 3:**
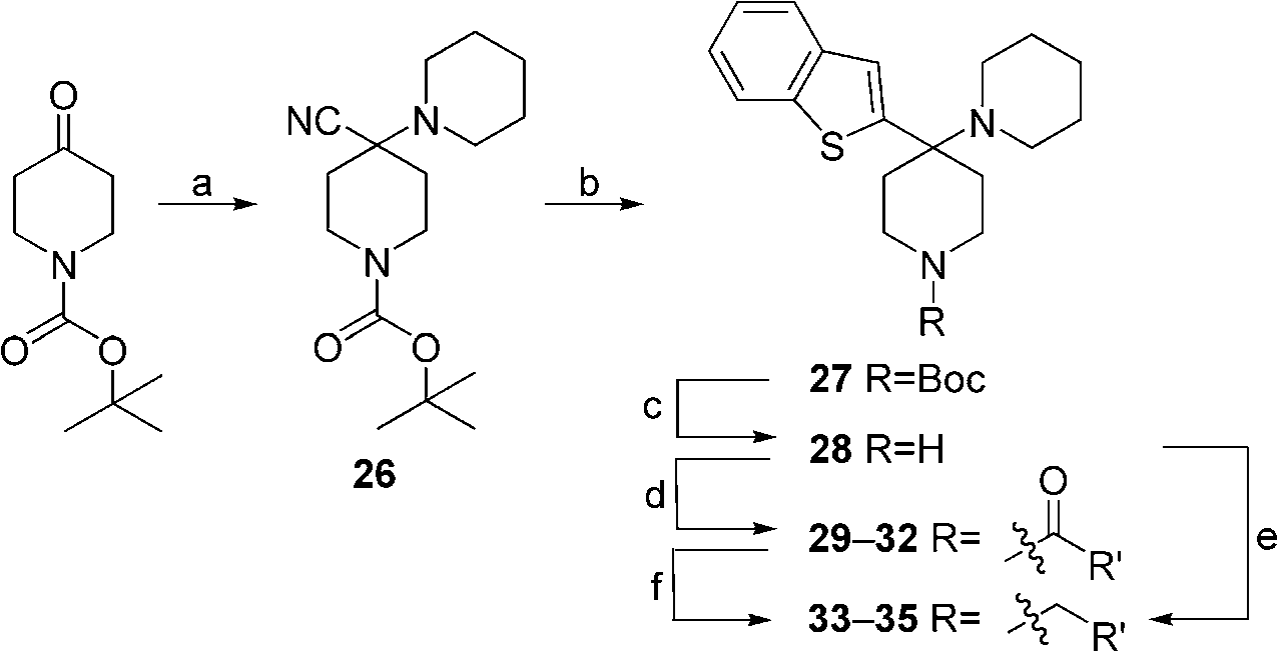
Synthesis of BTCP analogues containing a substituted nitrogen atom at the 4-position of the cyclohexyl moiety. See Table 2 for a list of R groups. *Reagents and conditions*: a) piperidine, acetone cyanohydrin, DMF, MgSO_4_, 50 °C, 4 d; b) benzo[*b*]thien-2-yl-MgBr, Et_2_O, 35 °C, 16 h; c) TFA, CH_2_Cl_2_, 0 °C, 1 h; d) R′COCl, DMAP, pyridine, 25 °C, 16 h; e) R′X, K_2_CO_3_, CH_3_CN, 82 °C, 16 h; f) LiAlH_4_, THF, 40 °C, 0.5–3 h.

**Scheme 4:**
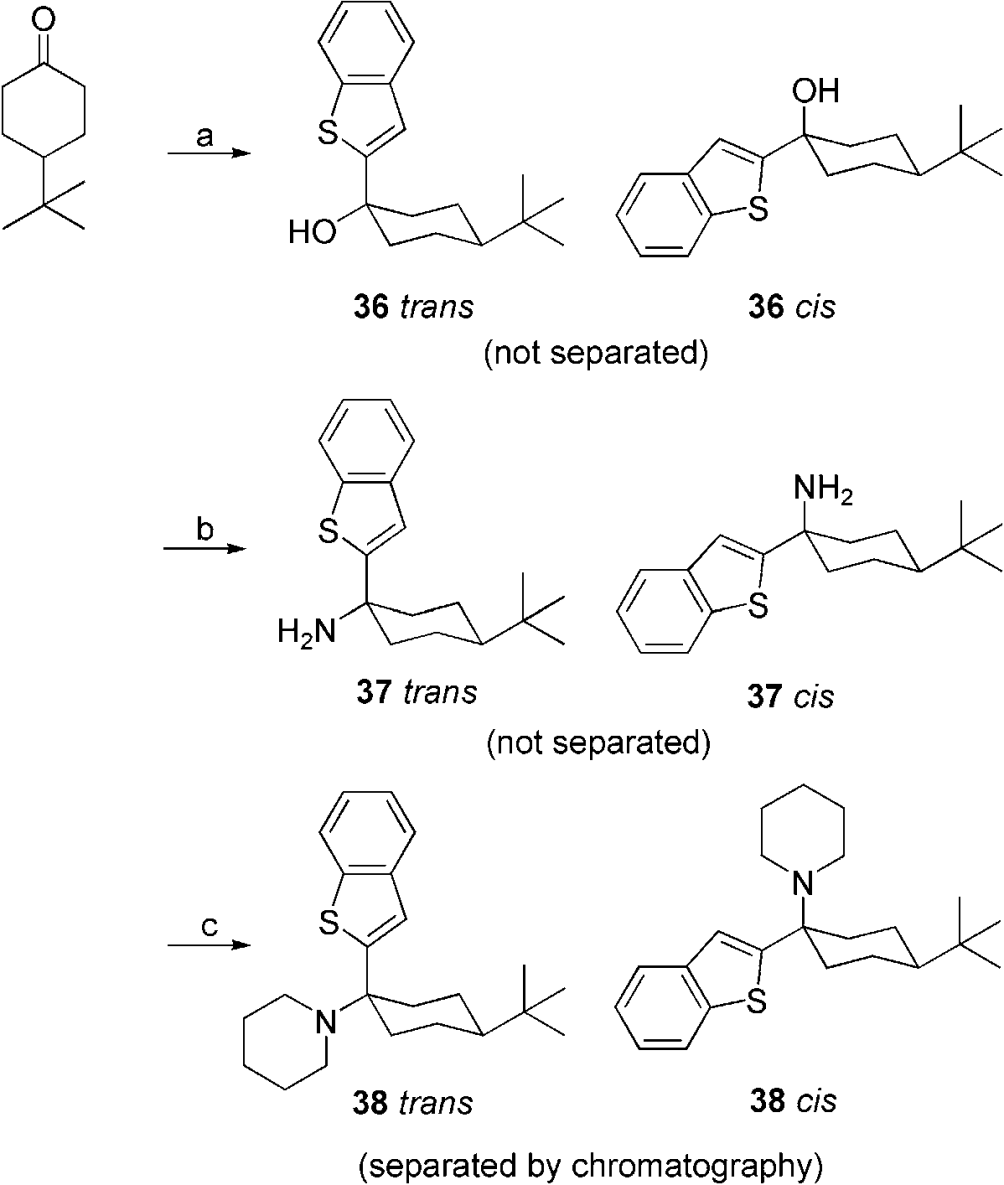
Synthesis of *cis* and *trans*-4-*tert*-butyl cyclohexyl BTCP analogues (**38**).9b *Reagents and conditions*: a) benzo[*b*]thien-2-yl-CeCl_2_, THF, −78→25 °C, 16 h; b) 1. TCA, NaN_3_, CHCl_3_, −25→0 °C, 55 min; 2. LiAlH_4_, THF, 25 °C, 2 h; c) 1,5-dibromopentane, K_2_CO_3_, CH_3_CN, 82 °C, 3.5 d.

Two strategies were employed to functionalise the 4-position of the cyclohexane moiety; first, preparation of a bipiperidinyl analogue (**28**), with subsequent derivatisation of the nitrogen atom, allowing the synthesis of a number of analogues with a minimal number of synthetic transformations (Scheme 3); and second, a stepwise preparation of *cis* and *trans* **38** containing a *tert*-butyl substitution at C4 of the cyclohexane ring (Scheme 4).

In order to prepare the bipiperidinyl **28** it was necessary to employ a suitable protecting group for the nitrogen atom. Previously it has been reported that both the benzyl and benzoyl nitrogen protecting groups are unsuitable for the preparation of substituted phencyclidines.9a Therefore, the Boc protecting group was employed during the Bruylants reaction giving the key protected intermediate **27** (Scheme 3). The Boc group of **27** was deprotected under acidic conditions to yield the secondary amine **28**, which subsequently underwent either acylation or alkylation reactions to give the substituted analogues **29**–**33**. However, the alkylation reactions proved problematic leading to the formation of significant quantities of quaternary ammonium salts as side products, which proved difficult to separate from the tertiary amines by column chromatography. Therefore, LiAlH_4_ reduction of the amide analogues **30** and **32** was used to prepare the tertiary amine analogues **34** and **35**, respectively.

Analogues **27**–**35** were assayed for their ability to inhibit *T. brucei* TryR as described above and the results are displayed in Table 2. The free amine **28** was approximately equal in activity to BTCP (**1**) (IC_50_=5.1 μm vs 3.3 μm), suggesting that the increased activity of the *N*-methyl analogue **18** is derived from the introduction of the methyl group, not through the introduction of a hydrogen bond donor. However, analogues containing larger hydrophobic amide or alkyl substitutions (analogues **29**–**31** & **34**) all possessed reduced inhibitory activity (IC_50_=6.6–19 μm). Similarly the Boc protected precursor **27** proved to be completely inactive in the TryR assay (IC_50_>100 μm). This demonstrates that the 4-position of the cyclohexane ring of BTCP (**1**) is not fully occluded by TryR upon inhibitor binding, but that the protein region around this position does not form favourable hydrophobic interactions. This conclusion is supported by the fact that analogues **32** and **33** containing polar substitutions were found to be approximately equipotent with BTCP (**1**) (IC_50_=2.6 μm and 4.4 μm, respectively vs 3.3 μm), and of similar potency to the *N*-methyl analogue **18**. Analogue **35** was found to be inactive in the TryR assay inconsistent with the results observed for **32** and **33**. However, this lack of activity could be due to **35** being the only analogue to contain three highly basic atoms.

**Table 2:** Substituted analogues of BTCP (**1**) and their inhibitory activities against *T. brucei* TryR.^[a]^

Compd	R^[b]^	IC_50_ [μm]	*T. brucei* EC_50_ [μm]	MRC5 EC_50_ [μm]
**27**	Boc	>100	ND	ND
**28**	H	5.1	2.5	11
**29**	COCH_3_	6.6	ND	ND
**30**	COPh	13	6.5	>50
**31**	COCH_2_Ph	12	4.3	>50
**32**	COCH_2_N(CH_3_)_2_	2.6	13	18
**33**	CH_2_CH_2_-*N*-Morpholine	4.4	20	>50
**34**	CH_2_Ph	19	15	>50
**35**	CH_2_CH_2_N(CH_3_)_2_	>100	ND	ND

[a] ND=not determined. [b] For full structures see Scheme 3.

Analogues **28** and **30**–**34** were assayed against *T. brucei* parasites and MRC-5 cells (Table 2). With the exception of compound **32**, all of the analogues showed some degree of selectivity against the parasites (>2-fold). However, as observed with the *N*-methyl analogue **16**, compounds **28**, **30**, **31** and **34** showed improved potency in the *T. brucei* assay over the enzyme assay. This is suggestive of either selective uptake, or an off-target effect for these analogues.

Analogues containing alkyl substitutions at C4 of the cyclohexyl ring have been previously prepared by employing either the Bruylants reaction (Scheme 1, route B), or in a stepwise sequence from tertiary benzylic alcohols (e.g. **36**) (Scheme 4). It has been demonstrated that the Bruylants reaction gives only a single isomer (*cis*) when 4-substituted α-aminonitriles are used as the substrates for the reaction.^17^ However, there was an interest in assaying both isomers of **38** as they have been shown to possess a different pharmacological selectivity^18^ and could offer an insight into the optimal arrangement of the piperidine ring, aromatic group and 4-cyclohexyl substituent relative to each other for the inhibition of TryR. Therefore, in order to access both isomers, a modification of the published synthetic route outlined in Scheme 4 was employed. The two isomers, *cis*- and *trans*-**38**, were separated by column chromatography at the final step. It has been demonstrated that the *cis* isomer elutes first when the mixture is purified with silica as the stationary phase.9b

*Cis*- and *trans*-**38** were assayed for their ability to inhibit TryR under the standard assay conditions and found to have IC_50_ values of >100 μm and 3.6 μm, respectively. This demonstrates that there is an absolute requirement for the piperidine moiety to be equatorial and conversely for the aromatic moiety to be in an axial conformation in order for BTCP analogues to inhibit TryR. Additionally, these results show that substituting BTCP with a bulky *tert*-butyl group at the 4-position of the cyclohexane ring leads to no appreciable change in TryR inhibitory activity (3.6 μm vs 3.3 μm for **1**), supporting the conclusion that the 4-position is not occluded by the protein structure upon binding of the inhibitor with TryR. *Trans*-**38** was also screened in the cell assay and found to have an EC_50_ value of 3.2 μm against *T. brucei* and inactive against the mammalian cell line (EC_50_>15 μm), again comparable to **1**.

## Conclusions

The investigations reported herein have confirmed that analogues of BTCP (**1**) represent a new class of TryR inhibitors, which are to our knowledge structurally distinct from inhibitors previously reported in the literature. Enzyme and cellular assays have demonstrated that analogues of this series are competitive inhibitors with respect to the natural TryR substrate, trypanothione, and that the analogues are marginally more potent against trypanosomes than mammalian cells in culture.

**Figure 2:**
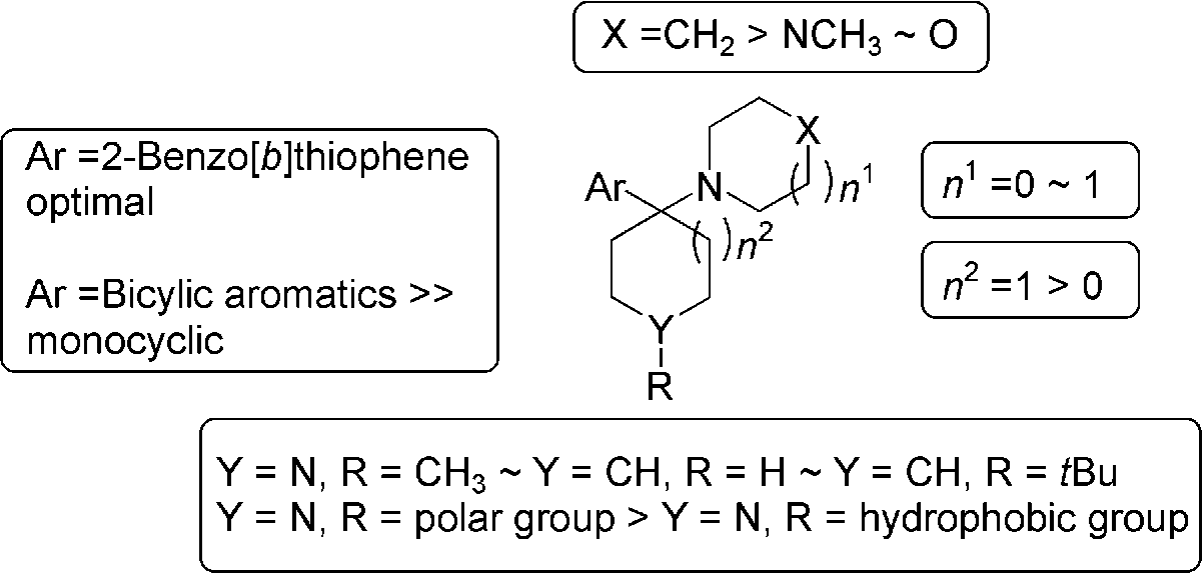
SAR summary for the inhibition of *T. brucei* TryR by BTCP (**1**) analogues.

Synthesis and screening of a diverse analogue collection has allowed a detailed SAR to be established for all moieties of the arylcyclohexylamine pharmacophore (Figure 2). However, although the essential structural features for maintaining the inhibitory activity of BTCP analogues have been determined, no functional group changes that significantly increase the potency against TryR have been identified.

From the rough correlation between *T. brucei* TryR IC_50_ and *T. brucei* EC_50_ values it is expected that TryR inhibitors in the single nanomolar range will be a requisite for adequate inhibition of parasite growth. However, given the preliminary SAR this goal is unlikely to be realised without the aid of a protein–ligand structure to identify potentially beneficial binding interactions. However, no noncovalent protein–ligand structures have been reported for TryR. Although it has been demonstrated that submicromolar inhibitors of TryR can be developed,^19^ these inhibitors are not considered druglike (e.g. MW>500). This requirement for high molecular weight compounds to efficiently inhibit TryR may be a direct consequence of TryR possessing a large, solvent-exposed active site.^20^ To date, druglike molecules have only achieved potencies in the low micromolar range,^6^ unfortunately this remains true for the BTCP series.

## Experimental Section

### Biology

#### TryR enzyme assay

**Figure 3:**
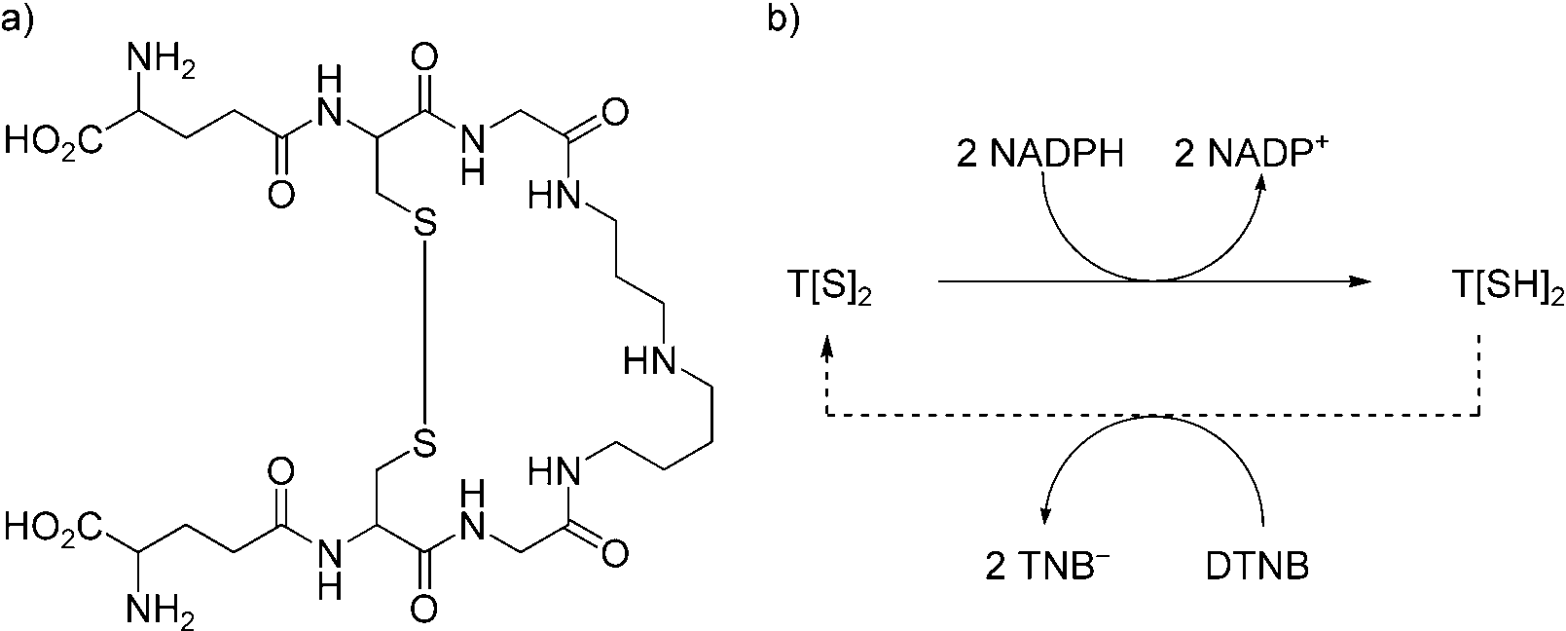
a) The structure of trypanothione (T[S]_2_), the substrate of TryR. b) The principle of the DTNB-coupled assay for TryR.

A nonenzymatically coupled assay for detecting TyrR activity was used.^10^ In this assay, the activity of TyrR is coupled to the reduction of DTNB (5,5′-dithiobis-(2-nitrobenzoic acid)) to 2TNB^−^ by dihydrotrypanothione (T[SH]_2_). Formation of 2TNB^−^ is measured as an increase in absorbance at 412 nm (Figure 3). The TyrR screening assay was miniaturised and optimised to a 384-well plate format. Assessment of the assay for robustness in an automated environment yielded the following typical performance statistics: Z′=0.84±0.001; %CV (plate)=3.65±0.4; signal to background=10±0.25; clomipramine IC_50_=12.4±0.14 μm.

Potency was determined as independent duplicates for all compounds tested. Serial titrations (10 half log increments) of test compounds from 30 μm to 1 nm were created in DMSO using the Janus automated 8 channel pipettor (Perkin–Elmer). A serial titration of clomipramine was used as a positive control in each assay plate; BTCP was used as an additional control in some screening plates. Using a Platemate Plus (Thermofisher Scientific), 500 nL of each test compound was transferred into assay plates (384 clear polystyrene plates) along with standard inhibitor and DMSO in the appropriate control wells. A TryR/DTNB/TrySH mixture (37.5 μL in buffer containing 40 mm Hepes and 1 mm Na_4_EDTA, pH 7.4) was then added to each well (Platemate Plus, Thermofisher Scientific) such that final assay concentrations were 3 nm, 50 μm and 6 μm, respectively. The reaction was started by addition of 4 μL NADPH (4 μL buffer for LO controls), to yield a final assay concentration 150 μm. The reaction was incubated for 35 min at room temperature. The absorbance was then measured at 405±8 nm using the Envision plate reader (Perkin–Elmer).

ActivityBase from IDBS was used for all data processing and analysis. Database querying and report creation was undertaken using SARgen version 5.4 and SARview version 6.1 from IDBS.

#### Cell-based assays

Trypanosomes (*T. b. brucei*, BSF 427 vsg221) were seeded in 96-well plates at 2000 cells per well in a volume of 200 μL of HMI-9T^21^ containing 10 % FCS. MRC-5 cells were seeded at 2000 cells per well in a volume of 200 μL of DMEM containing 10 % FCS and allowed to adhere for 24 h prior to use.

For compound assessment, compounds were serially diluted in 100 % DMSO through a ten-point, one in three dilution curve, in row orientation using a Janus 8 channel Varispan. This produced a working stock of 200× final concentration in the assay. Compound plates contained six test compounds and one standard compound occupying columns 1–10: row A was omitted from screening due to potential edge effect and row H contained the standard compound. Each compound working stock (1 μL) was then stamped into replicate clear 96-well polystyrene assay plates using a Platemate 2×2 (Matrix-Thermofisher) to achieve the final assay concentration at DMSO level of 0.5 %.

Assay plates (200 μL final volume per well) were incubated for 69 h at 37 °C in an atmosphere of 5 % CO_2_. Resazurin (20 μL of 500 μm) was then added to each well and the plates incubated for another 4 h. Plates were read for fluorescence at an excitation wavelength of 528 nm and an emission wavelength of 590 nm.

#### Mode of inhibition studies

An assay mixture consisting of TryR, NADPH and DTNB was made up in 40 mm HEPES; 1 mm EDTA (pH 7.4). Aliquots of the assay mixture (180 μL) containing three different concentrations of test compound were added to three rows of a microtitre plate, a fourth row contained only the assay mixture. The test compound concentration ranged from ∼0.25 to 1 times the IC_50_ value. Trypanothione disulphide was serially diluted across a fifth row of the plate to produce a 12-point range from 500 μm to 5.8 μm. The assay was initiated by transferring 20 μL of trypanothione disulphide row to each of the assay rows. The final 200 μL assay contained 150 μm NADPH; 50 μm DTNB and 20 mU mL^−1^ TryR. The linear rate of increase in absorbance at 412 nm was determined using a Molecular Devices Thermomax plate reader. Each data set was fitted by nonlinear regression to the Michaelis–Menten equation using GraFit 5.0 (Erithacus software). The resulting individual fits were examined as Lineweaver–Burke transformations and the graphs inspected for diagnostic inhibition patterns. The entire dataset was then globally fitted to the appropriate equation (competitive, mixed or uncompetitive inhibition).

### Chemistry

***General***: Chemicals and solvents were purchased from the Aldrich Chemical Company, Fluka, ABCR, VWR, Acros, Fisher Chemicals and Alfa Aesar and were used as received unless otherwise stated. Air and moisture sensitive reactions were carried out under an inert atmosphere of Ar in oven-dried glassware. Analytical thin-layer chromatography (TLC) was performed on precoated TLC plates (0.20 mm silica gel_60_ with fluorescent indicator UV 254) (Merck). Plates were air-dried and visualised under a UV lamp (UV254/365 nm), and where necessary, stained with a solution of ninhydrin or iodine on silica to aid identification. Flash column chromatography was performed using prepacked silica gel cartridges (230–400 mesh, 40–63 μm) (SiliCycle) using a Teledyne ISCO Combiflash Companion or Combiflash Retrieve. ^1^H NMR, ^13^C NMR, and 2D-NMR spectra were recorded on a Bruker Avance DPX 500 spectrometer (^1^H at 500.1 MHz, ^13^C at 125.8 MHz). Chemical shifts (*δ*) are expressed in ppm recorded using the residual solvent as the internal reference in all cases. Signal splitting patterns are described as singlet (s), doublet (d), triplet (t), quartet (q), multiplet (m), broad (br), or a combination thereof. Coupling constants (*J*) are quoted to the nearest 0.5 Hz. LC/MS analyses were performed with either an Agilent HPLC 1100 series connected to a Bruker Daltonics MicrOTOF, or an Agilent Technologies 1200 series HPLC connected to an Agilent Technologies 6130 quadrupole LC/MS; both instruments were connected to an Agilent diode array detector. LC/MS chromatographic separations were conducted with a Phenomenex Gemini C18 column, 50×3.0 mm, 5 μm particle size; mobile phase: H_2_O/CH_3_CN+0.1 % HCO_2_H, 80:20→5:95 over 3.5 min, and then held for 1.5 min; flow rate 0.5 mL min^−1^. High resolution electrospray measurements were performed on a Bruker Daltonics MicrOTOF mass spectrometer.

#### Procedures for the synthesis of BTCP analogues

**Method A (compounds 3, 5, 9**–**15, 17)**:^11^ *n*BuLi (1.6 m in hexanes, 4 eq) was added to a solution of the corresponding heteroaromatic compound (4 eq) in anhyd THF (10 mL) at −78 °C and stirred for 1 h. The resultant ArLi solution was then added via a cannula to an ice-cooled solution of the relevant benzotriazoyl adduct prepared by stirring the corresponding enamine (1 eq) and benzotriazole (1 eq) in anhyd Et_2_O (5 mL) for 1 h. The reaction was allowed to warm to RT and stirred for 16 h. Workup was initiated by the addition of aq citrate (10 % *w*/*v*, 20 mL), the layers separated and the organic layer further extracted with aq citrate (10 % *w*/*v*, 3×20 mL). The combined aqueous layers were basified to pH 10 (2 m aq NaOH), extracted into CH_2_Cl_2_ (4×50 mL) and the combined CH_2_Cl_2_ layers dried (MgSO_4_), filtered and concentrated in vacuo. The crude product was purified by flash column chromatography (EtOAc/Hexane, 0:100→60:40) and if necessary by trituration of the HCl salts from Et_2_O.

**Method B1 (compounds 4, 6 & 7)**:^14^ To a suspension of Mg turnings (1 eq) and I_2_ (2 mg, cat.) in anhyd Et_2_O (10 mL) was slowly added a solution of the relevant ArBr (1 eq) in anhyd Et_2_O (10 mL) and the mixture refluxed for 2–3 h. To the resultant Grignard solution was added a solution of nitrile **20** (1 eq) in anhyd Et_2_O (10 mL) and the reaction heated at reflux for 16 h. The reaction was worked up and the products purified as described for method A above.

**Method B2 (compounds 16, & 18)**:^15^ To a suspension of Mg turnings (27.5 mmol, 669 mg) in anhyd Et_2_O (25 mL) in a reflux apparatus was slowly added a solution of 1,2-dibromoethane (27.5 mmol, 5.17 g) in anhyd Et_2_O (25 mL) and the resultant mixture allowed to stir for 3 h. To the resultant MgBr_2_ solution was added a solution of benzo[*b*]thien-2-yl-lithium (27.5 mmol) prepared as outlined in method A above and the reaction stirred for 30 min at RT. The generated Grignard solution was then slowly added to the relevant nitrile (10 mmol, 2.07 g) in anhyd Et_2_O (10 mL) and the reaction heated to reflux for 16 h. The reaction was worked up and the products purified as described for method A above.

**BTCP 1**: This was purchased from Tocris Bioscience as the maleate salt. LCMS analysis confirmed compound identity and that purity was >95 % (diode array).

**1-(1-Phenylcyclohexyl)piperidine (PCP) 2**: 1-(1-Piperidino)cyclohexene (1 mmol, 165 mg) was added to a suspension of 1*H*-benzotriazole (1 mmol, 119 mg) in anhyd Et_2_O (5 mL) and stirred at RT for 1 h. The reaction mixture was then cooled to 0 °C prior to the addition of phenyllithium (1.8 m in dibutylether, 4 mmol, 2.22 mL). The reaction mixture was allowed to warm to RT and stirred for 16 h. The reaction was worked up and purified as described for method A above to give a clear gum (36 mg, 15 %), which was further purified by trituration of the HCl salt from Et_2_O. The reported analysis is for the HCl salt. ^1^H NMR (500 MHz, CD_3_OD): *δ*=1.20–1.31 (3 H, m, CH_2_C*H*_2_CH_2_ & 1×C*H*H), 1.34–1.43 (1 H, m, C*H*H), 1.63–1.68 (1 H, m, C*H*H), 1.75–1.95 (9 H, m, 2×CCH*H*, 1×C*H*H & 3×CH_2_), 2.37–2.44 (2 H, m, 2×NCH*H*), 3.09–3.13 (2 H, m, 2×CC*H*H), 3.76–3.80 (2 H, m, 2×NC*H*H), 7.55–7.63 (3 H, m, 2 *m*-PhH & *p*-PhH), 7.70 ppm (2 H, d, *J*=7.5 Hz, 2×*o*-PhH). ^13^C NMR (125 MHz, CD_3_OD): *δ*=23.1 (CH_2_), 24.0 (CH_2_), 24.8 (CH_2_), 26.2 (CH_2_), 32.4 (C*C*H_2_), 48.9 (NCH_2_) 72.9 (C), 130.5 (Ph CH), 131.0 (Ph CH), 131.1 (Ph CH), 131.7 ppm (Ph C). MS (LCMS ES+): *m*/*z* (%) 159 (14) [*M*−Piperidine]^+^, 244 (100) [*M*+H]^+^. HRMS (ES+): calcd for C_17_H_26_N_1_ [*M*+H]^+^ 244.2060, found 244.2059 (0.28 ppm).

**1-(1-Thiophen-2-yl)cyclohexyl)piperidine 3**: Prepared by method A from thiophene (4 mmol, 337 mg) and 1-(1-piperidino)cyclohexene (1 mmol, 165 mg). The product was obtained as an brown oil (158 mg, 63 %). The reported analysis is for the HCl salt. ^1^H NMR (500 MHz, CD_3_OD): *δ*=1.30–1.45 (4 H, m, CH_2_C*H*_2_CH_2_ & 2×C*H*H), 1.66–1.68 (1 H, m, C*H*H), 1.78–1.81 (1 H, m, C*H*H), 1.88–2.03 (8 H, m, 2×CCH*H* & 3×CH_2_), 2.55–2.59 (2 H, m, 2×NCH*H*), 2.87–2.89 (2 H, m, 2×CC*H*H), 3.78–3.80 (2 H, m, 2×NC*H*H), 7.27 (1 H, dd, *J*=5.0, 4.0 Hz, thiophene H4), 7.44 (1 H, dd, *J*=4.0, 1.0 Hz, thiophene H3), 7.76 ppm (1 H, dd, *J*=5.0, 1.0 Hz, thiophene H5). ^13^C NMR (125 MHz, CD_3_OD): *δ*=23.1 (CH_2_), 24.3 (CH_2_), 24.8 (CH_2_), 25.6 (CH_2_), 34.7 (CCH_2_), 48.85 [under CD_3_OD, identified by DEPT135 & HSQC] (NCH_2_), 71.3 (C), 126.2 (thiophene C4), 130.1 (thiophene C5), 132.5 (thiophene C3), 136.8 ppm (thiophene C2). MS (LCMS ES+): *m*/*z* (%) 165 (50) [*M*−Piperidine]^+^, 250 (100) [*M*+H]^+^. HRMS (ES+): calcd for C_15_H_24_N_1_S_1_ [*M*+H]^+^ 250.1624, found 250.1622 (0.89 ppm).

**1-(1-Biphenyl-4-yl)cyclohexyl)piperidine 4**: Prepared by method B1 from 4-bromobiphenyl (15.5 mmol, 3.65 g). The product was obtained as a colourless crystalline solid (1.04 g, 21 %). The reported analysis is for the free base. ^1^H NMR (500 MHz, CDCl_3_): *δ*=1.28–1.40 (4 H, m, 2×CH_2_C*H*_2_CH_2_), 1.45–1.57 (6 H, m, 2×CH_2_ & 2×C*H*H), 1.73–1.80 (2 H, m, 2×C*H*H), 2.00–2.07 (2 H, m, 2×CC*H*H), 2.16–2.22 (2 H, m, 2×CC*H*H), 2.27–2.39 (2 H, m, 2×NCH_2_), 7.35–7.39 (3 H, m, AA′BB′& *p*-PhH), 7.47 (2 H, t, *J*=8.0 Hz, 2 *m*-PhH), 7.60–7.61 (2 H, m, AA′BB′), 7.66 ppm (2 H, dd, *J*=8.0, 1.0 Hz, 2×*o*-PhH). ^13^C NMR (125 MHz, CDCl_3_): *δ*=22.5 (CH_2_), 25.0 (CH_2_), 26.5 (CH_2_), 27.2 (CH_2_), 33.7 (C*C*H_2_), 46.5 (NCH_2_), 60.9 (C), 126.05 (biphenyl CH), 127.0 (biphenyl CH), 127.1 (biphenyl CH), 127.8 (biphenyl CH), 128.7 (biphenyl CH), 138.6 (biphenyl C), 139.1 (biphenyl C), 140.9 ppm (biphenyl C). MS (LCMS ES+): *m*/*z* (%) 320 (100) [*M*+H]^+^. HRMS (ES+): calcd for C_23_H_30_N_1_ [*M*+H]^+^ 320.2373, found 320.2375 (−0.68 ppm).

**1-(1-Benzo[*b*]furan-2-yl)cyclohexyl)piperidine 5**: Prepared by method A from benzo[*b*]furan (4 mmol, 473 mg) and 1-(1-piperidino)cyclohexene (1 mmol, 165 mg). The product was obtained as a yellow oil (239 mg, 84 %). The reported analysis is for the HCl salt. ^1^H NMR (500 MHz, CD_3_OD): *δ*=1.31–1.40 (4 H, m, CH_2_C*H*_2_CH_2_ & 2×C*H*H), 1.69–2.00 (10 H, m, 2×CCH*H* & 4×CH_2_), 2.66–2.70 (2 H, m, 2×NCH*H*), 2.99–3.01 (2 H, m, 2×CC*H*H), 3.83–3.85 (2 H, m, 2×NC*H*H), 7.33 (1 H, d, *J*=0.5 Hz, benzo[*b*]furan H3, 7.35–7.39 (1 H, m, benzo[*b*]furan H), 7.44–7.48 (1 H, m, benzo[*b*]furan H), 7.62 (1 H, dd, *J*=8.5, 1.0 Hz, benzo[*b*]furan H), 7.75–7.76 ppm (1 H, m, benzo[*b*]furan H). ^13^C NMR (125 MHz, CD_3_OD): *δ*=22.9 (CH_2_), 24.4 (CH_2_), 24.8 (CH_2_), 25.5 (CH_2_), 31.9 (C*C*H_2_), 49.53 [under CD_3_OD, identified by DEPT135 & HSQC] (NCH_2_), 70.2 (C), 112.6 (benzo[*b*]furan CH), 112.9 (benzo[*b*]furan C3), 123.1 (benzo[*b*]furan CH), 124.9 (benzo[*b*]furan CH), 127.2 (benzo[*b*]furan CH), 128.8 (benzo[*b*]furan C), 150.4 (benzo[*b*]furan C), 156.5 ppm (benzo[*b*]furan C). MS (LCMS ES+): *m*/*z* (%) 199 (82) [*M*−piperidine]^+^, 284 (100) [*M*+H]^+^. HRMS (ES+): calcd for C_19_H_26_N_1_O_1_ [*M*+H]^+^ 284.2009, found 284.2008 (0.26 ppm).

**1-(1-Naphthalen-1-yl)cyclohexyl)piperidine 6**: Prepared by method B1 from 1-bromonaphthalene (10 mmol, 2.07 g). The product was obtained as a clear oil (316 mg, 11 %). The reported analysis is for the HCl salt. Note, NMR analysis acquired at 50 °C. ^1^H NMR (500 MHz, CD_3_OD): *δ*=1.12–1.52 (4 H, m, 2×CH_2_C*H*_2_CH_2_), 1.52–1.57 (1 H, m, C*H*H), 1.75–1.80 (1 H, m, CH*H*), 1.86–1.95 (6 H, m, 2×CH_2_ & 2×C*H*H), 2.18–2.25 (2 H, m, 2×CC*H*H), 2.75–2.84 (2 H, m, 2×NC*H*H), 3.27–3.76 (2 H, m, 2×CC*H*H), 3.82–3.87 (2 H, m, 2×NC*H*H), 7.61 (1 H, dd, *J*=8.0, 8.0 Hz, naphthyl H), 7.65–7.70 (2 H, m, 2×naphthyl H), 8.01 (1 H, d, *J*=7.5 Hz, naphthyl H), 8.06 (1 H, dd, *J*=8.0, 1.0 Hz, naphthyl H), 8.10 (1 H, d, *J*=8.5 Hz, naphthyl H), 8.62 ppm (1 H, d, *J*=9.0, naphthyl H). ^13^C NMR (125 MHz, CDCl_3_): *δ*=21.6 (CH_2_), 23.0 (CH_2_), 23.7 (CH_2_), 24.7 (CH_2_), 34.2 (C*C*H_2_), 49.1 (NCH_2_), 76.2 (C), 124.4 (naphthyl CH), 124.7 (naphthyl CH), 125.5 (naphthyl CH), 126.7 (naphthyl C), 127.2 (naphthyl CH), 130.3 (naphthyl CH), 132.1 (naphthyl CH), 132.3 (naphthyl CH), 133.1 (naphthyl C), 135.6 ppm (naphthyl C). MS (LCMS ES+): *m*/*z* (%) 294 (100) [*M*+H]^+^. HRMS (ES+): calcd for C_21_H_28_N_2_ [*M*+H]^+^ 294.2216, found 294.2225 (2.97 ppm).

**1-(1-Naphthalen-2-yl)cyclohexyl)piperidine 7**: Prepared by method B1 from 2-bromonaphthalene (10 mmol, 2.07 g). The product was obtained as a white crystalline solid (191 mg, 7 %). The reported analysis is for the HCl salt. ^1^H NMR (500 MHz, CD_3_OD): *δ*=1.19–1.35 (3 H, m, CH_2_C*H*_2_CH_2_ & CH*H*), 1.38–1.47 (1 H, m, CH*H*), 1.63–1.67 (1 H, m, C*H*H), 1.72–1.76 (1 H, m, C*H*H), 1.83–1.95 (6 H, m, 2×CH_2_ & 2×C*H*H), 2.01–2.07 (2 H, m, 2×CC*H*H), 2.43–2.50 (2 H, m, 2×CCH*H*), 3.23–3.28 (2 H, m, 2×NC*H*H), 3.83–3.88 (2 H, m, 2×CCH*H*), 7.61–7.67 (2 H, m, naphthyl H-6 & H-7), 7.78 (1 H, dd, *J*=9.0, 1.5 Hz, naphthyl H-3), 7.98–7.80 (1 H, m, naphthyl H), 8.05–8.06 (1 H, m, naphthyl H), 8.09 (1 H, d, *J*=9.0 Hz, naphthyl H-4), 8.25 ppm (1 H, s, naphthyl H-1). ^13^C NMR (125 MHz, CD_3_OD): *δ*=23.1 (CH_2_), 24.1 (CH_2_), 24.8 (CH_2_), 26.2 (CH_2_), 32.6 (C*C*H_2_), 49.0 [under CD_3_OD, identified by DEPT135 & HSQC] (NCH_2_), 73.1 (C), 127.0 (naphthyl C3), 128.0 (naphthyl CH), 128.5 (naphthyl CH), 128.9 (naphthyl CH), 129.1 (naphthyl C), 129.8 (naphthyl CH), 130.1 (naphthyl C4), 131.8 (naphthyl C1), 134.6 (naphthyl C), 134.9 ppm (naphthyl C). MS (LCMS ES+): *m*/*z* (%) 294 (100) [*M*+H]^+^. HRMS (ES+): calcd for C_21_H_28_N_2_ [*M*+H]^+^ 294.2216, found 294.2220 (1.39 ppm).

**1-Methyl-2-(1-piperidin-1-yl)cyclohexyl)-1*H*-indole 8**: Prepared by a modification of method B2 from 1-methylindole (4 mmol, 525 mg) and nitrile **20** (4 mmol, 768 mg). The product was obtained as a white solid (297 mg, 25 %). The reported analysis is for the free base. ^1^H NMR (500 MHz, CDCl_3_): *δ*=.1.18–1.28 (1 H, m, CH_2_C*H*HCH_2_), 1.42–1.57 (8 H, m, CH_2_C*H*_2_CH_2_, 2×CH_2_ & 2×C*H*H, 1.63–1.71 (3 H, m, 3×C*H*H), 1.83–1.90 (2 H, m, 2×CC*H*H), 2.22–2.27 (2 H, m, 2×CCH*H*), 2.55–2.57 (4 H, m, 2×NCH_2_), 4.09 (3 H, s, CH_3_), 6.52 (1 H, s, indole H3), 7.10 (1 H, ddd, *J*=7.5, 7.5, 1.0 Hz, indole H5), 7.20 (1 H, ddd, *J*=8.0, 7.5, 1.0 Hz, indole H6), 7.32 (1 H, d, *J*=8.0 Hz, indole H7), 7.57 (1 H, d, *J*=7.5 Hz, indole H-4). ^13^C NMR (125 MHz, CDCl_3_): *δ*=23.8 (CH_2_), 25.5 (CH_2_), 26.6 (CH_2_), 27.3 (CH_2_), 32.3 (CH_3_), 46.8 (NCH_2_), 62.3 (C), 104.6 (indole CH), 108.9 (indole CH), 119.0 (indole CH), 119.8 (indole CH), 120.8 (indole CH), 127.0 (indole C), 138.7 (indole C), 142.5 ppm (indole C) [Note, two of the CH_2_ carbons have an identical chemical shift]. MS (LCMS ES+): *m*/*z* (%) 212 (100) [*M*−piperidine]^+^. HRMS (ES+): calcd for C_20_H_29_N_2_ [*M*+H]^+^ 297.2325, found 297.2313 (4.16 ppm).

**1-(1-Benzo[*b*]thiazol-2-yl)cyclohexyl)piperidine 9**: Prepared by method A from benzo[*b*]thiazole (4 mmol, 541 mg) and 1-(1-piperidino)cyclohexene (1 mmol, 165 mg). The product was obtained as a yellow semisolid (164 mg, 55 %). The reported analysis is for the free base. ^1^H NMR (500 MHz, CDCl_3_): *δ*=1.33–1.37 (2 H, m, CH_2_), 1.44–1.57 (8 H, m, 2×CH*H* & 3×CH_2_), 1.74–1.81 (2 H, m, 2×CH*H*), 2.07–2.20 (4 H, m, 2×CCH_2_), 2.50–2.57 (4 H, m, 2×NCH_2_), 7.36 (1 H, dd, *J*=7.5, 7.5 Hz, benzo[*b*]thiazole H6), 7.45 (1 H, dd, *J*=7.5, 7.5 Hz, benzo[*b*]thiazole H5), 7.88 (1 H, d, *J*=7.5 Hz, benzo[*b*]thiazole H7), 8.03 ppm (1 H, d, *J*=7.5 Hz, benzo[*b*]thiazole H4) ^13^C NMR (125 MHz, CDCl_3_): *δ*=22.4 (CH_2_), 25.0 (CH_2_), 26.0 (CH_2_), 27.1 (CH_2_), 34.7 (C*C*H_2_), 46.9 (NCH_2_), 63.7 (C), 121.4 (benzo[*b*]thiazole C7), 123.0 (benzo[*b*]thiazole C4), 124.6 (benzo[*b*]thiazole C6), 125.5 (benzo[*b*]thiazole C5), 135.0 (benzo[*b*]thiazole C7a), 152.9 (benzo[*b*]thiazole C3a), 176.0 ppm (benzo[*b*]thiazole C2). MS (LCMS ES+): *m*/*z* (%) 301 (100) [*M*+H]^+^. HRMS (ES+): calcd for C_18_H_25_N_2_S_1_ [*M*+H]^+^ 301.1733, found 301.1718 (4.80 ppm).

**1-(1-Benzo[*b*]thiophen-3-yl)cyclohexyl)piperidine 10 & 1-(1-(3-Bromo-benzo[*b*]thiophen-2-yl)cyclohexyl)piperidine 11**: Prepared by method A from 3-bromobenzo[*b*]thiophene (4 mmol, 852 mg) and 1-(1-piperidino)cyclohexene (1 mmol, 165 mg). The reaction gave two products that could be separated by column chromatography. The reported analysis is for the free bases.

**For 10**: *R*_f_=0.20 (EtOAc/hexanes, 1:1), clear oil (143 mg, 48 %). ^1^H NMR (500 MHz, CDCl_3_): *δ*=1.24–1.30 (1 H, m, CH_2_CH*H*CH_2_), 1.36–1.49 (8 H, C*H*_2_CH_2_C*H*_2_ & CH*H*C*H*_2_CH*H*), 1.617–1.71 (3 H, m, CH_2_C*H*HCH_2_ & C*H*HCH_2_C*H*H), 1.93–1.99 (2 H, m, 2×CCH*H*), 2.30–2.35 (2 H, m, 2×CCH*H*), 2.52–2.58 (4 H, m, 2×NCH_2_), 7.27 (1 H, s, benzo[*b*]thiophene H2), 7.30–7.33 (2 H, m, benzo[*b*]thiophene H5 & H6), 7.84–7.87 (1 H, m, benzo[*b*]thiophene H7), 8.55–8.59 ppm (1 H, m, benzo[*b*]thiophene H4). ^13^C NMR (125 MHz, CDCl_3_): *δ*=23.7 (CH_2_), 25.6 (CH_2_), 26.7 (CH_2_), 27.4 (CH_2_), 30.8 (C*C*H_2_), 47.1 (NCH_2_), 63.5 (C), 122.5 (benzo[*b*]thiophene C7), 122.7 (benzo[*b*]thiophene CH), 123.6 (benzo[*b*]thiophene CH), 124.2 (benzo[*b*]thiophene C2), 126.8 (benzo[*b*]thiophene C4), 138.5 (benzo[*b*]thiophene C), 140.5 benzo[*b*]thiophene C), 140.8 ppm (benzo[*b*]thiophene C). MS (LCMS ES+): *m*/*z* (%) 215 (100) [*M*−piperidine]^+^, 300 (34) [*M*+H]^+^. HRMS (ES+): calcd for C_19_H_26_N_1_S_1_ [*M*+H]^+^ 300.1780, found 300.1767 (4.50 ppm).

**For 11**: *R*_f_=0.49 (EtOAc/hexanes, 1:1), clear oil (58 mg, 15 %). ^1^H NMR (500 MHz, CDCl_3_): *δ*=1.20–1.26 (2 H, m, CH_2_), 1.35–1.49 (8 H, m, 3×CH_2_ & 2×C*H*H), 1.64–1.73 (2 H, m, 2×C*H*H), 1.93–2.00 (2 H, m, 2×CC*H*H), 2.49–2.62 (6 H, m, 2×CCH*H* & 2×NCH_2_), 7.27–7.36 (2 H, m, benzo[*b*]thiophene H5 & H6), 7.67 (1 H, d, *J*=8.0 Hz), benzo[*b*]thiophene H), 7.80 ppm (1 H, d, *J*=8.0 Hz, benzo[*b*]thiophene H). MS (LCMS ES+): *m*/*z* (%) 293 (57) [^79^Br *M*−piperidine]^+^, 295 (57) [^81^Br *M*−piperidine]^+^, 378 (100) [^79^Br *M*+H]^+^, 380 (100) [^81^Br *M*+H]^+^. HRMS (ES+): calcd for C_19_H_25_^79^Br_1_N_1_S_1_ [*M*+H]^+^ 378.0886, found 378.0897 (−2.99 ppm).

**1-(1-(5-Bromo-benzo[*b*]thiophen-2-yl)cyclohexyl)piperidine 12**: Prepared by method A from 5-bromobenzo[*b*]thiophene (4 mmol, 852 mg) and 1-(1-piperidino)cyclohexene (1 mmol, 165 mg). The product was obtained as a white solid (43 mg, 11 %). The reported analysis is for the HCl salt. ^1^H NMR (500 MHz, CD_3_OD): *δ*=1.31–1.41 (2 H, m, 2×C*H*H), 1.42–1.51 (2 H, m, CH_2_C*H*_2_CH_2_), 1.68–1.73 (1 H, m, C*H*H), 1.76–1.82 (1 H, m, C*H*H), 1.84–1.93 (2 H, m, 2×C*H*H), 1.95–2.07 (6 H, 2×CH_2_ & 2×C*H*H), 2.69–2.76 (2 H, m, 2×NC*H*H), 2.91–2.95 (2 H, m, 2×CC*H*H), 3.82–3.87 (2 H, m, 2×NC*H*H), 7.62 (1 H, dd, *J*=8.5, 2.0 Hz, benzo[*b*]thiophene H6), 7.75 (1 H, s, benzo[*b*]thiophene H3), 7.92 (1 H, d, *J*=8.5 Hz, benzo[*b*]thiophene H7), 8.15 ppm (1 H, d, *J*=2.0 Hz, benzo[*b*]thiophene H4). ^13^C NMR (125 MHz, CDCl_3_): *δ*=23.0 (CH_2_), 24.3 (CH_2_), 24.9 (CH_2_), 25.4 (CH_2_), 34.5 (C*C*H_2_), 49.4 (NCH_2_), 71.4 (C), 120.0 (benzo[*b*]thiophene C5), 125.1 (benzo[*b*]thiophene C7), 128.3 (benzo[*b*]thiophene C4), 129.4 (benzo[*b*]thiophene C3), 130.2 (benzo[*b*]thiophene C6), 139.6 (benzo[*b*]thiophene C), 140.3 (benzo[*b*]thiophene C), 142.3 ppm (benzo[*b*]thiophene C). MS (LCMS ES+): *m*/*z* (%) 293 (70) [^79^Br *M*−piperidine]^+^, 295 (74) [^81^Br *M*−piperidine]^+^, 378 (98) [^79^Br *M*+H]^+^, 380 (100) [^81^Br *M*+H]^+^. HRMS (ES+): calcd for C_19_H_25_^79^BrN_1_S_1_ [*M*+H]^+^ 378.0886, found 378.0872 (3.47 ppm).

**1-(1-Benzo[*b*]thiophen-2-yl)cyclohexyl)pyrrolidine 13**: Prepared by method A from benzothiophene (537 mg) and 1-(1-pyrrolidino)cyclohexene (151 mg). The product was obtained as an orange solid (149 mg, 52 %). The reported analysis is for the HCl salt. ^1^H NMR (MHz, CD_3_OD): *δ*=1.37–1.44 (1 H, m, C*H*H), 1.46–1.55 (2 H, m, 2×C*H*H), 1.69–1.74 (1 H, m, C*H*H), 1.85–1.99 (6 H, m, 2×NCH_2_C*H*_2_, 2× C*H*H), 2.03–2.09 (2 H, m, 2×CC*H*H), 2.79–2.85 (2 H, m, 2×CC*H*H), 3.37–3.43 (2 H, m, 2×NC*H*H), 3.54–3.59 (2 H, m, 2×NC*H*H), 7.48–7.52 (2 H, m, 2×benzo[*b*]thiophene H), 7.83 (1 H, s, benzo[*b*]thiophene H3), 7.96–8.01 ppm (2 H, m, 2×benzo[*b*]thiophene H). ^13^C NMR (125 MHz, CD_3_OD): *δ*=23.9 (CH_2_), 25.5 (CH_2_), 35.8 (C*C*H_2_), 49.5 [under CD_3_OD, identified by DEPT135 & HSQC] (NCH_3_), 123.4 (benzo[*b*]thiophene CH), 125.8 (benzo[*b*]thiophene CH), 126.3 (benzo[*b*]thiophene CH), 127.2 (benzo[*b*]thiophene C3), 130.1 (benzo[*b*]thiophene CH), 137.4 (benzo[*b*]thiophene C), 140.7 (benzo[*b*]thiophene C), 141.5 ppm (benzo[*b*]thiophene C). MS (LCMS ES+): *m*/*z* (%) 215 (100) [*M*−pyrrolidine]^+^, 286 (41) [*M*+H]^+^. HRMS (ES+): calcd for C_18_H_24_N_1_S_1_ [*M*+H]^+^ 286.1624, found 286.1616 (2.74 ppm).

**1-(1-Benzo[*b*]thiophen-2-yl)cyclohexyl)diethylamine 14**: Prepared by method A from benzo[*b*]thiophene (4 mmol, 537 mg) and 1-(1-diethylamino)cyclohexene (1 mmol, 153 mg).13a The product was obtained as a yellow oil (17 mg, 4 %). The reported analysis is for the HCl salt. ^1^H NMR (500 MHz, CD_3_OD): *δ*=1.36–1.41 (7 H, 2×CH_3_ & CH_2_C*H*HCH_2_), 1.46–1.55 (2 H, m, 2×CH*H*), 1.68–1.74 (1 H, m, CH_2_C*H*HCH_2_), 1.93–1.99 (2 H, m, 2×C*H*H), 2.03–2.10 (2 H, m, 2×CC*H*H), 2.89–3.01 (4 H, 2×NC*H*H & 2×CC*H*H), 3.77–3.85 (2 H, m, 2×NC*H*H), 7.48–7.52 (2 H, m, 2×benzo[*b*]thiophene H), 7.85 (1 H, s, benzo[*b*]thiophene H3), 7.95–8.00 ppm (2 H, m, 2×benzo[*b*]thiophene H). ^13^C NMR (125 MHz, CD_3_OD): *δ*=12.7 (CH_3_), 24.2 (CH_2_), 25.4 (CH_2_), 34.8 (C*C*H_2_), 47.2 (NCH_2_), 73.1 (C), 123.3 (benzo[*b*]thiophene CH), 125.7 (benzo[*b*]thiophene CH), 126.2 (benzo[*b*]thiophene CH), 127.3 (benzo[*b*]thiophene CH), 130.3 (benzo[*b*]thiophene C3), 140.6 (benzo[*b*]thiophene C), 141.5 ppm (benzo[*b*]thiophene C). [Note, one quaternary carbon is missing, or two carbons have identical shifts]. MS (LCMS ES+): *m*/*z* (%) 215 (100) [*M*−diethylamine]^+^. HRMS (ES+): calcd for C_18_H_26_N_1_S_1_ [*M*+H]^+^ 288.1780, found 288.1784 (−1.24 ppm).

**1-(1-Benzo[*b*]thiophen-2-yl)cyclohexyl)morpholine 15**: Prepared by method A from benzo[*b*]thiophene (4 mmol, 537 mg) and 1-(1-morpholino)cyclohexene (1 mmol, 167 mg). The product was obtained as a yellow solid (203 mg, 67 %). The reported analysis is for the HCl salt. ^1^H NMR (500 MHz, CD_3_OD): *δ*=1.37–1.43 (1 H, m, C*H*H), 1.45–1.55 (2 H, m, CH_2_), 1.69–1.74 (1 H, m, CH*H*), 1.96–2.01 (2 H, m, 2×C*H*H), 2.07–2.13 (2 H, m, 2×CC*H*H), 2.88–2.93 (2 H, m, 2×CC*H*H), 2.99–3.05 (2 H, m, 2×OC*H*H), 3.66–3.71 (2 H, m, 2×OC*H*H), 3.88–3.94 (2 H, m, 2×NC*H*H), 4.04–4.08 (2 H, m, 2×NC*H*H), 7.49–7.53 (2 H, m, 2×benzo[*b*]thiophene H), 7.81 (1 H, s, benzo[*b*]thiophene H3), 7.97–8.01 ppm (2 H, m, 2×benzo[*b*]thiophene H). ^13^C NMR (125 MHz, CD_3_OD): *δ*=24.3 (CH_2_), 25.5 (CH_2_), 34.1 (C*C*H_2_), 48.1 (OCH_2_), 65.2 (NCH_2_), 72.1 (C), 123.4 (benzo[*b*]thiophene CH), 125.9 (benzo[*b*]thiophene CH), 126.3 (benzo[*b*]thiophene CH), 127.3 (benzo[*b*]thiophene CH), 130.5 (benzo[*b*]thiophene C3), 140.7 (benzo[*b*]thiophene C), 141.7 ppm (benzo[*b*]thiophene C). MS (LCMS ES+): *m*/*z* (%) 215 (100) [*M*−morpholine]^+^, 302 (12) [*M*+H]^+^. HRMS (ES+): calcd for C_18_H_24_N_1_O_1_S_1_ [*M*+H]^+^ 302.1573, found 302.1562 (3.71 ppm).

**1-(1-Benzo[*b*]thiophen-2-yl)cyclohexyl)-4-methylpiperazine 16**: Prepared by method B2 from benzo[*b*]thiophene (27.5 mmol, 3.69 g) and nitrile **21** (10 mmol, 2.07 g). The product was obtained as a clear oil (12 mg, 0.4 %). The reported analysis is for the free base. ^1^H NMR (500 MHz, CDCl_3_): *δ*=1.44–1.51 (4 H, m, cyclohexyl CH_2_C*H*_2_CH_2_ & 2×C*H*H), 1.73–1.79 (2 H, m, 2×C*H*H), 2.01–2.14 (4 H, m, 2×CCH_2_), 2.27 (3 H, s, CH_3_), 2.45–2.67 (8 H, 4×piperazine CH_2_), 7.09 (1 H, s, benzo[*b*]thiophene H3), 7.25–7.32 (2 H, m, benzo[*b*]thiophene H5 & H6), 7.70 (1 H, dd, *J*=7.5, 1.0 Hz, benzo[*b*]thiophene H7), 7.75–7.76 ppm (1 H, m, benzo[*b*]thiophene H4). ^13^C NMR (125 MHz, CDCl_3_): *δ*=22.4 (CH_2_), 25.9 (CH_2_), 35.1 (C*C*H_2_), 44.9 (NCH_2_), 45.6 (CH_3_), 55.9 (NCH_2_), 60.7 (C), 121.4 (benzo[*b*]thiophene C3), 121.9 (benzo[*b*]thiophene CH), 123.1 (benzo[*b*]thiophene CH), 123.7 (benzo[*b*]thiophene CH), 123.9 (benzo[*b*]thiophene CH), 139.0 (benzo[*b*]thiophene C), 139.6 (benzo[*b*]thiophene C), 147.7 ppm (benzo[*b*]thiophene C). MS (LCMS ES+): *m*/*z* (%) 215 (62) [*M*−piperazine]^+^, 315 (100) [*M*+H]^+^. HRMS (ES+) calcd for C_19_H_27_N_2_S_1_ [*M*+H]^+^ 315.1889, found 315.1882 (2.46 ppm).

**1-(1-Benzo[*b*]thiophen-2-yl)cyclopentyl)piperidine 17**: Prepared by method A from benzo[*b*]thiophene (4 mmol, 537 mg) and 1-(1-piperidino)cyclopentene (1 mmol, 151 mg). The product was obtained as a yellow semisolid (28 mg, 10 %). The reported analysis is for the HCl salt. ^1^H NMR (500 MHz, CD_3_OD): *δ*=1.15–1.24 (1 H, m, CH_2_C*H*HCH_2_), 1.55–1.68 (3 H, m, 2×C*H*H & CH_2_C*H*HCH_2_), 1.80–1.93 (6 H, m, 2×C*H*H & C*H*_2_CH_2_C*H*_2_), 2.18–2.24 (2 H, m, 2×CC*H*H), 2.74–2.85 (4 H, m, 2×CC*H*H & 2×NC*H*H), 3.59–3.64 (2 H, m, 2×NC*H*H), 7.33–7.37, 2 H, m, 2×benzo[*b*]thiophene CH), 7.69 (1 H, s, benzo[*b*]thiophene H3), 7.80–7.85 ppm (2 H, m, 2×benzo[*b*]thiophene CH). ^13^C NMR (125 MHz, CD_3_OD): *δ*=22.7 (CH_2_), 22.9 (CH_2_), 24.6 (CH_2_), 38.2 (C*C*H_2_), 52.3 (NCH_2_), 77.3 (C), 123.3 (benzo[*b*]thiophene CH), 125.7 (benzo[*b*]thiophene CH), 126.2 (benzo[*b*]thiophene CH), 127.1 (benzo[*b*]thiophene CH), 130.1 (benzo[*b*]thiophene C3), 138.0 (benzo[*b*]thiophene C), 140.8 (benzo[*b*]thiophene C), 141.6 ppm (benzo[*b*]thiophene C). MS (LCMS ES+): *m*/*z* (%) 86 (100) [piperidine+H]^+^, 201 (82) [*M*−piperidine]^+^. HRMS (ES+): calcd for C_18_H_24_N_1_S_1_ [*M*+H]^+^ 286.1624, found 286.1619 (1.81 ppm).

**4′-Benzo[*b*]thiophen-2-yl)-1′-methyl-1,4’bipiperidine 18**: Prepared by method B2 from benzo[*b*]thiophene (27.5 mmol, 3.69 g) and nitrile **22** (10 mmol, 2.07 g). The product was obtained as a white solid (87 mg, 3 %). The reported analysis is for the free base. ^1^H NMR (500 MHz, CDCl_3_): *δ*=1.30–1.35 (2 H, m, CH_2_C*H*_2_CH_2_), 1.52–1.57 (4 H, m, C*H*_2_CH_2_C*H*_2_), 2.23–2.27 (4 H, m, 2×CCH_2_), 2.29 (3 H, s, CH_3_), 2.36–2.46 (6 H, m, 2NCH_2_ & 2×CH_3_NC*H*H), 2.72–2.76 (2 H, m, 2×CH_3_NC*H*H), 7.04 (1 H, s, benzo[*b*]thiophene H3), 7.28–7.35 (2 H, m, benzo[*b*]thiophene H5 & H6), 7.74 (1 H, d, *J*=7.5 Hz, benzo[*b*]thiophene H7), 7.80 ppm (1 H, d, *J*=8.0, benzo[*b*]thiophene H4). ^13^C NMR (125 MHz, CDCl_3_): *δ*=24.9 (CH_2_*C*H_2_CH_2_), 27.0 (*C*H_2_CH_2_*C*H_2_), 35.0 (C*C*H_2_), 45.8 (CH_3_), 46.6 (NCH_2_), 51.9 (CH_3_N*C*H_2_), 58.8 (C), 120.9 (benzo[*b*]thiophene C3), 122.0 (benzo[*b*]thiophene C4), 123.2 (benzo[*b*]thiophene C7), 123.8 (benzo[*b*]thiophene CH), 124.0 (benzo[*b*]thiophene CH), 138.9 (benzo[*b*]thiophene C), 139.6 (benzo[*b*]thiophene C), 147.0 ppm (benzo[*b*]thiophene C). MS (LCMS ES+): *m*/*z* (%) 230 (100) [*M*−piperidine]^+^, 315 (9) [*M*+H]^+^. HRMS (ES+): calcd for C_19_H_27_N_2_S_1_ [*M*+H]^+^ 315.1889, found 315.1882 (2.35 ppm).

**1-(2-Benzo[*b*]thiophen-2-yl)propan-2-yl)piperidine 19**: Prepared by method A from benzo[*b*]thiophene (8 mmol, 1.07 g) and 1-(prop-1-en-2-yl)piperidine13b (2 mmol, 250 mg). The product was obtained as a yellow oil (79 mg, 15 %). The reported analysis is for the free base. ^1^H NMR (500 MHz, CDCl_3_): *δ*=1.41–1.46 (2 H, m, CH_2_C*H*_2_CH_2_), 1.47 (6 H, s, 2×CH_3_), 1.55–1.59 (4 H, m, C*H*_2_CH_2_C*H*_2_), 2.48–2.55 (4 H, m, 2×NCH_2_), 7.04 (1 H, s, benzo[*b*]thiophene H3), 7.23–7.31 (2 H, m, benzo[*b*]thiophene H5 & H6), 7.66 (1 H, d, *J*=7.5 Hz, benzo[*b*]thiophene H7), 7.78 ppm (1 H, d, *J*=8.0 Hz, benzo[*b*]thiophene H4). ^13^C NMR (125 MHz, CDCl_3_): *δ*=25.0 (CH_2_*C*H_2_CH_2_), 25.2 (CH_3_), 26.8 (*C*H_2_CH_2_*C*H_2_), 47.7 (NCH_2_), 59.7 (C), 118.4 (benzo[*b*]thiophene C3), 122.2 (benzo[*b*]thiophene C4), 122.8 (benzo[*b*]thiophene C7), 123.5 (benzo[*b*]thiophene CH), 123.7 (benzo[*b*]thiophene CH), 139.8 (benzo[*b*]thiophene C), 139.9 (benzo[*b*]thiophene C), 158.9 ppm (benzo[*b*]thiophene C). MS (LCMS ES+): *m*/*z* (%) 175 [*M*−piperidine]^+^, 260 [*M*+H]^+^. HRMS (ES+): calcd for C_16_H_22_N_1_S_1_ [*M*+H]^+^ 260.1467, found 260.1458 (3.77 ppm).

**α-Amino nitriles (20, 21 & 22)**: Prepared following the α-amino nitrile synthesis described in reference 15 and used without further purification.

**Phenyl(1-(piperidin-1-yl)cyclohexyl)methanone 23**: To a solution of nitrile **20** (5 mmol, 960 mg) in anhyd Et_2_O (25 mL) at −78 °C was slowly added phenyllithium (6 mmol, 1.8 m solution in dibutylether, 3.33 mL) over 30 min. The reaction was then allowed to warm to 4 °C and stirred for 16 h. Aq HCl (10 %, 20 mL) was then added to the reaction and the reaction further stirred for 30 min at 0 °C. The reaction was then diluted with EtOAc (50 mL), the layers separated and the organic layer extracted with aq citrate (10 %, 2×50 mL). The combined aqueous layers were basified to pH 10 (NH_4_OH) and extracted with CH_2_Cl_2_ (4×100 mL). The combined CH_2_Cl_2_ layers were dried (MgSO_4_), filtered and concentrated in vacuo. The resultant crude product was purified by flash column chromatography (EtOAc/Hexane, 0:100→2:98) to give a yellow semisolid (237 mg, 17 %). The reported analysis is for the HCl salt. ^1^H NMR (500 MHz, CD_3_OD): *δ*=0.95–1.05 (2 H, m, cyclohexyl C*H*HCH_2_C*H*H), 1.15–1.24 (1 H, m, cyclohexyl CH_2_C*H*HCH_2_), 1.38–1.49 (2 H, m, cyclohexyl CH_2_C*H*HCH_2_ & piperidinyl CH_2_C*H*HCH_2_), 1.64–1.69 (2 H, m, cyclohexyl C*H*HCH2C*H*H), 1.73–1.93 (7 H, m, 2×CC*H*H & piperidinyl C*H*_2_C*H*HC*H*_2_), 2.65–2.70 (2 H, m, 2×CC*H*H), 3.10–3.16 (2 H, m, 2×NC*H*H), 3.63–3.67 (2 H, m, 2×NC*H*H), 7.47 (2 H, t, *J*=8.0 Hz, 2 *m*-PhH), 7.55–7.58 (1 H, m, *p*-PhH), 7.65–7.68 ppm (2 H, m, 2×*o*-PhH). ^13^C NMR (125 MHz, CD_3_OD): *δ*=23.0 (CH_2_), 23.8 (CH_2_), 24.9 (CH_2_), 25.3 (CH_2_), 31.7 (C*C*H_2_), 51.1 (NCH_2_), 77.2 (C), 128.6 (*o*-Ph CH), 130.2 (*m*-Ph CH), 133.9 (*p*-Ph CH), 141.2 (Ph C), 203.6 ppm (CO). MS (LCMS ES+): *m*/*z* (%) 272 (100) [*M*+H]^+^. HRMS (ES+): calcd for C_18_H_26_N_1_O_1_ [*M*+H]^+^ 272.2009, found 272.2000 (3.23 ppm).

**Benzo[*b*]thiophen-2-yl(1-(piperidin-1-yl)cyclohexyl)methanone 24**: *n*BuLi (10 mmol, 1.6 m in hexanes, 6.25 mL) was added to a solution of benzo[*b*]thiophene (10 mmol, 1.34 g) in anhyd THF (25 mL) at −78 °C and stirred for 1 h. The resultant ArLi solution was added via a cannula to a solution of nitrile **20** (10 mmol, 1.92 g) in anhyd Et_2_O (20 mL) at 0 °C over 15 min and stirred for 5 h. Aq HCl (10 %, 20 mL) was then added to the reaction and the reaction further stirred for 30 min at 0 °C. The reaction was then diluted with EtOAc (50 mL), the layers separated and the organic layer extracted with aq HCl (1 m, 3×25 mL). The combined aqueous layers were basified to pH 10 (solid KOH) and extracted with CH_2_Cl_2_ (4×50 mL). The combined CH_2_Cl_2_ layers were dried (MgSO_4_), filtered and concentrated in vacuo. The resultant crude product was purified by flash column chromatography (EtOAc/Hexane, 0:100→10:90) to give an off-white foam (414 mg, 13 %). The reported analysis is for the free base. ^1^H NMR (500 MHz, CDCl_3_): *δ*=1.08–1.17 (1 H, m, C*H*H), 1.49–1.73 (13 H, m, C*H*H, 5×CH_2_ & 2×CC*H*H), 2.16–2.21 (2 H, m, 2×CC*H*H), 2.64–2.68 (4 H, m, 2×NCH_2_), 7.37 (1 H, ddd, *J*=8.0, 7.0, 1.0 Hz, benzo[*b*]thiophene H6), 7.43 (1 H, ddd, *J*=8.0, 7.0, 1.0 Hz, benzo[*b*]thiophene H5), 7.83–7.88 (2 H, m, benzo[*b*]thiophene H4 & H7), 8.25 ppm (1 H, d, *J*=0.5 Hz, benzo[*b*]thiophene H3). ^13^C NMR (125 MHz, CDCl_3_): *δ*=23.2 (CH_2_), 25.2 (CH_2_), 26.0 (CH_2_), 26.2 (CH_2_), 29.7 (C*C*H_2_), 47.6 (NCH_2_), 70.1 (C), 122.4 (benzo[*b*]thiophene C4), 124.4 (benzo[*b*]thiophene C6), 125.4 (benzo[*b*]thiophene C7), 126.7 (benzo[*b*]thiophene C5), 130.7 (benzo[*b*]thiophene C3), 138.0 (benzo[*b*]thiophene C), 138.9 (benzo[*b*]thiophene C), 143.3 (benzo[*b*]thiophene C), 199.1 ppm (CO). MS (LCMS ES+): *m*/*z* (%) 328 (100) [*M*+H]^+^. HRMS (ES+): calcd for C_20_H_26_N_1_O_1_S_1_ [*M*+H]^+^ 328.1730, found 328.1724 (1.77 ppm).

**Diphenyl(1-(piperidin-1-yl)cyclohexyl)methanol 25**: To a solution of ketone **20** (1 mmol, 271 mg) in anhyd Et_2_O (10 mL) at 0 °C was added phenyllithium (1 mmol, 1.8 m solution in dibutylether, 556 μL) and the reaction allowed to warm to 25 °C and stirred for 2.5 h. Workup was initiated by the addition of saturated aq NH_4_Cl (10 mL), the layers were separated and the aqueous phase further extracted with Et_2_O (3×10 mL), the combined organics were dried (MgSO_4_), filtered and concentrated in vacuo. The resultant crude product was purified by flash column chromatography (EtOAc/Hexane, 0:100→1:99) to give a white solid (239 mg, 68 %). The reported analysis is for the HCl salt. ^1^H NMR (500 MHz, CD_3_OD): *δ*=1.28–1.36 (1 H, m, CH_2_C*H*HCH_2_), 1.43–1.93 (11 H, m, 5×CH_2_ & CH_2_C*H*HCH_2_), 2.21–2.29 (2 H, m, 2×CC*H*H), 2.52–2.58 (2 H, m, 2×CC*H*H), 3.07–3.19 (4 H, m, 2×NCH_2_), 7.41 (2 H, t, *J*=7.5 Hz, 2× *p*-PhH), 7.49 (4 H, dd, *J*=7.5, 7.5 Hz, 4 *m*-PhH), 7.95 ppm (4 H, d, *J*=7.5 Hz, 4×*o*-PhH). ^13^C NMR (125 MHz, CD_3_OD): *δ*=22.7 (CH_2_), 23.5 (CH_2_), 25.2 (CH_2_), 27.0 (CH_2_), 29.7 (C*C*H_2_), 54.7 (NCH_2_), 80.6 (C), 83.0 (C), 128.8 (*o*-Ph CH), 129.5 (*p*-Ph CH), 129.7 (*m*-Ph CH), 143.5 ppm (Ph C). MS (LCMS ES+): *m*/*z* (%) 350 (100) [*M*+H]^+^. HRMS (ES+): calcd for C_24_H_32_N_1_O_1_ [*M*+H]^+^ 350.2478, found 350.2481 (0.83 ppm).

***tert*****-Butyl 4′-cyano-1,4′-bipiperidine-1′-carboxylate 26**: To a suspension of MgSO_4_ (126 mmol, 15.2 g) in anhyd DMF (6 mL) was added 1-Boc-4-piperidone (26 mmol, 5.18 g), piperidine (40 mmol, 3.41 g) and acetone cyanohydrin (26 mmol, 2.21 g). The reaction mixture was then heated to 50 °C for 4 d. The reaction mixture was then poured into ice water (100 mL) and stirred for 30 min before extraction with Et_2_O (4×100 mL). The combined Et_2_O layers were washed with water (5×500 mL), dried (MgSO_4_), filtered and concentrated in vacuo to give a cream solid that was used without further purification (6.53 g, 86 %). The reported analysis is for the free base. ^1^H NMR (500 MHz, CDCl_3_): *δ*=1.47 (9 H, s, *t*Bu), 1.49–1.53 (2 H, m, CH_2_C*H*_2_CH_2_), 1.61–1.72 (6 H, m, C*H*_2_CH_2_C*H*_2_ & 2×CC*H*H), 2.11–2.16 (2 H, m, 2×CC*H*H), 2.56–2.64 (4 H, m, 2×NCH_2_), 3.12–3.21 (2 H, m, 2×NBocC*H*H), 3.89–4.06 ppm (2 H, m, 2×NBocC*H*H). ^13^C NMR (125 MHz, CDCl_3_): *δ*=24.1 (CH_2_*C*H_2_CH_2_), 26.1 (*C*H_2_CH_2_*C*H_2_), 28.4 (*t*Bu CH_3_), 33.6 (C*C*H_2_) [broad peak due to restricted flexibility of the ring system], 39.6 & 40.4 (BocNCH_2_) [two peaks due to restricted flexibility of the ring system], 47.7 (NCH_2_), 60.5 (C), 80.0 (*t*Bu C), 118.3 (CN), 154.4 ppm (CO). MS (LCMS ES+): *m*/*z* (%) 211 (20) [*M*−*t*Bu−CN+H]^+^, 238 (64) [*M*−*t*Bu+H]^+^, 267 (18) [*M*−CN]^+^, 294 (100) [*M*+H]^+^. HRMS (ES+): calcd for C_16_H_28_N_3_O_2_ [*M*+H]^+^ 294.2176, found 294.2181 (−1.55 ppm).

***tert*****-Butyl 4′-(benzo[*b*]thiophen-2-yl)-1,4′-bipiperidine-1′-carboxylate 27**: Prepared by method B2 from benzo[*b*]thiophene (55 mmol, 7.38 g) and nitrile **26** (20 mmol, 5.87 g). The product was obtained as a white solid (465 mg, 6 %). The reported analysis is for the free base. ^1^H NMR (500 MHz, CDCl_3_): *δ*=1.31–1.35 (2 H, m, CH_2_C*H*_2_CH_2_), 1.47 (9 H, s, *t*Bu), 1.53–1.58 (4 H, m, C*H*_2_CH_2_C*H*_2_), 2.06–2.13 (2 H, m, 2×CC*H*H), 2.16–2.22 (2 H, m, 2×CC*H*H), 2.41–2.45 (4 H, m, 2×NCH_2_), 3.39–3.44 (2 H, m, 2×NBocC*H*H), 3.63–3.68 (2 H, m, 2×NBocC*H*H), 7.05 (1 H, s, benzo[*b*]thiophene H3), 7.29–7.36 (2 H, m, benzo[*b*]thiophene H5 & H6), 7.75 (1 H, d, *J*=7.5 Hz, benzo[*b*]thiophene H7), 7.81 ppm (1 H, d, *J*=7.5 Hz, benzo[*b*]thiophene H4). ^13^C NMR (125 MHz, CDCl_3_): *δ*=24.8 (CH_2_*C*H_2_CH_2_), 26.9 (*C*H_2_CH_2_*C*H_2_), 28.5 (*t*Bu CH_3_), 34.9 & 35.2 (C*C*H_2_) [two peaks due to restricted flexibility of the ring system], 39.5 & 40.5 (BocNCH_2_) [two peaks due to restricted flexibility of the ring system], 46.7 (NCH_2_), 59.3 (C), 79.4 (*t*Bu C), 120.8 (benzo[*b*]thiophene C3), 122.0 (benzo[*b*]thiophene C4), 123.3 (benzo[*b*]thiophene C7), 123.9 (benzo[*b*]thiophene CH), 124.1 (benzo[*b*]thiophene CH), 138.8 (benzo[*b*]thiophene C), 139.5 (benzo[*b*]thiophene C), 146.1 (benzo[*b*]thiophene C), 154.9 ppm (CO). MS (LCMS ES+): *m*/*z* (%) 260 (100) [*M*−piperidine−*t*Bu]^+^, 423 (16) [*M*+Na]^+^. HRMS (ES+): calcd for C_23_H_33_N_2_O_2_S_1_ [*M*+H]^+^ 401.2257, found 401.2257 (0.10 ppm).

**4′-(Benzo[*b*]thiophen-2-yl)-[1,4′]bipiperidine 28**: To a solution of **27** (0.43 mmol, 174 mg) in anhyd CH_2_Cl_2_ (10 mL) at 0 °C was added TFA (1 mL) and the reaction mixture stirred for 1 h, before being poured into aq NaOH (2 m, 10 mL). The resultant biphasic mixture was separated and the aqueous layer extracted with CH_2_Cl_2_ (3×10 mL), the CH_2_Cl_2_ layers were then combined, dried (MgSO_4_), filtered and concentrated in vacuo. The crude product was purified by flash column chromatography (CH_3_OH/CH_2_Cl_2_, 0:100→10:90) to give an off-white foam (75 mg, 58 %). The reported analysis is for the free base. ^1^H NMR (500 MHz, CDCl_3_): *δ*=1.28–1.34 (2 H, m, CH_2_C*H*_2_CH_2_), 1.52–1.56 (4 H, m, C*H*_2_CH_2_C*H*_2_), 2.19–2.26 (4 H, m, 2×CCH_2_), 2.37–2.43 (4 H, m, 2×NCH_2_), 2.89–2.93 (2 H, m, 2×NHC*H*H), 3.19–3.24 (2 H, m, 2×NHC*H*H), 7.03 (1 H, s, benzo[*b*]thiophene H3), 7.28–7.35 (2 H, m, benzo[*b*]thiophene H5 & H6), 7.74 (1 H, d, *J*=7.5 Hz, benzo[*b*]thiophene H7), 7.79 ppm (1 H, d, *J*=8.0 Hz, benzo[*b*]thiophene H4). ^13^C NMR (125 MHz, CDCl_3_): *δ*=24.7 (CH_2_*C*H_2_CH_2_), 26.8 (*C*H_2_CH_2_*C*H_2_), 34.8 (C*C*H_2_), 41.5 (NHCH_2_), 46.3 (NCH_2_), 58.9 (C), 120.8 (benzo[*b*]thiophene C3), 121.9 (benzo[*b*]thiophene C4), 123.2 (benzo[*b*]thiophene C7), 123.9 (benzo[*b*]thiophene CH), 124.1 (benzo[*b*]thiophene CH), 138.6 (benzo[*b*]thiophene C), 139.3 (benzo[*b*]thiophene C), 145.9 ppm (benzo[*b*]thiophene C). MS (LCMS ES+): *m*/*z* (%) 171.5 (70) [*M*+MeCN+2H]^2+^, 216 (100) [*M*−piperidine]^+^, 301 (20) [*M*+H]^+^.

**General acylation procedure for the synthesis of analogues 29**–**32**: TFA (1 mL) was added to a solution of **27** (0.125 or 0.25 mmol, 50 or 100 mg) in anhyd CH_2_Cl_2_ (9 mL), at 0 °C and stirred for 2 h before the reaction was concentrated in vacuo. The resultant crude secondary amine **28** was redissolved in anhyd pyridine (5 mL), before the addition of cat DMAP (1 mg) and the relevant acid chloride (4 eq) and the reaction mixture stirred at RT for 16 h. The reaction was concentrated in vacuo and the crude mixture partitioned between CH_2_Cl_2_ (5 mL) and aq NaOH (2 m, 5 mL) and further worked up and purified as described for **28** above.

**1-(4′-Benzo[*b*]thiophen-2-yl)-1,4′-bipiperidin-1′-yl)ethanone 29**: Prepared following the general acylation procedure using AcCl (1 mmol, 78.5 mg) to give a brown glass (58 mg, 68 %). The reported analysis is for the HCl salt. ^1^H NMR (500 MHz, CD_3_OD): *δ*=1.29–1.39 (1 H, m, CH_2_C*H*HCH_2_), 1.75–1.90 (3 H, m, C*H*HCH*H*C*H*H), 1.97–2.03 (2 H, m CH*H*CH_2_CH*H*), 2.05–2.12 (1 H, m, C*H*H), 2.15 (3 H, s, CH_3_), 2.17–2.24 (1 H, m, CH*H*), 2.63–2.70 (1 H, m, C*H*H), 2.73–2.82 (2 H, m, 2×CH*H*), 2.97–3.03 (2 H, m, 2×CH*H*), 3.15–3.22 (1 H, m, CH*H*), 3.81–3.87 (2 H, m, 2×CH*H*), 4.13–4.18 (1 H, m, CH*H*), 4.72–4.78 (1 H, m, CH*H*), 7.49–7.54 (2 H, m, 2×benzo[*b*]thiophene H), 7.88 (1 H, s, benzo[*b*]thiophene H3), 7.97–8.02 ppm (2 H, m, 2×benzo[*b*]thiophene H). MS (LCMS ES+): *m*/*z* (%) 258 (44) [*M*−piperidine]^+^, 343 (100) [*M*+H]^+^. HRMS (ES+): calcd for C_20_H_27_N_2_O_1_S_1_ [*M*+H]^+^ 343.1839, found 343.1836 (0.63 ppm).

**(4′-(Benzo[*b*]thiophen-2-yl)-1,4′-bipiperidinyl-1′-yl)(phenyl)methanone 30**: Prepared following the general acylation procedure using benzoyl chloride (1 mmol, 141 mg) to give a brown glass (77 mg, 76 %). The reported analysis is for the HCl salt. Note, peaks are broad and poorly defined, possibly due to rotamers, or restricted flexibility in the aliphatic ring systems. ^1^H NMR (500 MHz, CD_3_OD): *δ*=1.27–1.41 (1 H, m, CH_2_C*H*HCH_2_), 1.71–2.01 (5 H, m, C*H*_2_C*H*HC*H*_2_), 2.13–2.32 (2 H, m, 2×CH*H*), 2.65–3.17 (6 H, m, 2×CH_2_, & 2×C*H*H), 3.67–4.04 (3 H, m, 3×C*H*H), 4.77–4.89 (1 H, m, C*H*H), 7.45–7.56 (7 H, m, 5×PhH & 2×benzo[*b*]thiophene H), 7.87 (1 H, s, benzo[*b*]thiophene H3), 7.97–8.02 ppm (2 H, m, 2×benzo[*b*]thiophene H). MS (LCMS ES+): *m/z* (%) 320 (70) [*M*-piperidine]^+^, 405 (100) [*M*+H]^+^ HRMS (ES+): calcd for C_25_H_29_N_2_O_1_S_1_ [*M*+H]^+^ 405.1995, found 405.1981 (3.44 ppm).

**1-(4′-(Benzo[*b*]thiophen-2-yl)-1,4′-bipiperidinyl-1′-yl)-2-phenylethanone 31**: Prepared following the general acylation procedure using phenylacetyl chloride (0.5 mmol, 77 mg) to give a clear glass (40 mg, 76 %). The reported analysis is for the HCl salt. ^1^H NMR (500 MHz, CD_3_OD): *δ*=1.27–1.37 (1 H, m, CH_2_C*H*HCH_2_), 1.72–2.04 (7 H, m, C*H*_2_CH*H*C*H*_2_ & 2×C*H*H), 2.67–2.78 (3 H, m, 3×C*H*H), 2.90–2.96 (1 H, m, C*H*H), 2.98–3.04 (1 H, m, C*H*H), 3.10–3.17 (1 H, m, C*H*H), 3.73–3.90 (4 H, m, 2×C*H*H & COC*H*_2_Ph), 4.25–4.31 (1 H, m, C*H*H), 4.77–4.83 (1 H, m, C*H*H), 7.27–7.32 (3 H, m, 2×*o*-PhH & *p*-PhH), 7.35–7.39 (2 H, m, 2 *m*-PhH), 7.49–7.54 (2 H, m, 2×benzo[*b*]thiophene CH), 7.85 (1 H, s, benzo[*b*]thiophene H3), 7.96–8.02 ppm (2 H, m, 2×benzo[*b*]thiophene CH). MS (LCMS ES+): *m*/*z* (%) 419 (100) [*M*+H]^+^. HRMS (ES+): calcd for C_26_H_31_N_2_O_1_S_1_ [*M*+H]^+^ 419.2152, found 419.2140 (2.67 ppm).

**1-(4′-(Benzo[*b*]thiophen-2-yl)-1,4′-bipiperidinyl-1′-yl)-2-(dimethylamino)ethanone 32**: Prepared following the general acylation procedure using dimethylaminoacetyl chloride hydrochloride (1 mmol, 158 mg) to give an orange glass (27 mg, 28 %). The reported analysis is for the HCl salt. ^1^H NMR (500 MHz, CD_3_OD): *δ*=1.29–1.38 (1 H, m, CH_2_CH*H*CH_2_), 1.75–1.80 (1 H, m, CH_2_CH*H*CH_2_), 1.95–2.02 (4 H, m, C*H*_2_CH_2_C*H*_2_), 2.32–2.38 (1 H, C*H*H, m,), 2.47–2.54 (1 H, m, C*H*H), 2.74–2.80 (3 H, m, 2×NC*H*H & C*H*H), 2.93 (3 H, s, CH_3_), 2.98–3.05 (5 H, m, CH_3_ & 2×C*H*H), 3.14–3.21 (1 H, m, C*H*H), 3.82–3.94 (3 H, m, 2×NC*H*H & C*H*H), 4.22 (1 H, d, *J*=16.0 Hz, COC*H*H), 4.49 (1 H, d, *J*=16.0 Hz, COC*H*H), 4.71–4.76 (1 H, m, C*H*H), 7.49–7.54 (2 H, m, 2×benzo[*b*]thiophene CH), 7.90 (1 H, s, benzo[*b*]thiophene H3), 7.97–8.02 ppm (2 H, m, 2×benzo[*b*]thiophene CH). MS (LCMS ES+): *m*/*z* (%) 151 (41) [*M*+H−piperidine]^2+^, 193.5 (17) [*M*+2H]^2+^, 301 (100) [*M*−piperidine]^+^, 386 (7) [*M*+H]^+^. HRMS (ES+): calcd for C_22_H_32_N_3_O_1_S_1_ [*M*+H]^+^ 386.2261, found 386.2270 (−2.45 ppm).

**4-(2-(4′-(Benzo[*b*]thiophen-2-yl)-1,4′-bipiperidin-1′-yl)ethyl)morpholine 33**: TFA (1 mL) was added to a solution of **27** (0.25 mmol, 100 mg) in anhyd CH_2_Cl_2_ (10 mL), at 0 °C and stirred for 1.5 h before being poured into aq NaOH (2 m, 10 mL). The resultant biphasic mixture was separated and the aqueous layer extracted with CH_2_Cl_2_ (3×10 mL), the CH_2_Cl_2_ layers were then combined, dried (MgSO_4_), filtered and concentrated in vacuo. The resultant crude secondary amine **28** was redissolved in anhyd CH_3_CN (5 mL), before the addition of K_2_CO_3_ (0.375 mmol, 52 mg) and 4-(2-chloroethyl)morpholine hydrochloride (0.5 mmol, 93 mg) and the reaction mixture stirred at 82 °C for 4 d. The reaction was then filtered and the reaction mixture adsorbed directly onto silica and purified as described for **28** to give a clear glass (16 mg, 15 %). The reported analysis is for the HCl salt. Note, peaks are broad and poorly defined making assignment of the spectra difficult. ^1^H NMR (500 MHz, CD_3_OD): *δ*=1.32–1.41 (1 H, m, CH*H*), 1.76–1.82 (1 H, m, CH*H*), 1.89–2.05 (4 H, m), 2.77–2.88 (4 H, m), 2.77–2.88 (4 H, m), 2.99–3.11 (2 H, m), 3.21–3.68 (10 H, m) [Note, overlaps solvent peak], 3.83–4.03 (8 H, m), 7.51–7.57 (2 H, m, 2×benzo[*b*]thiophene H), 7.94 (1 H, s, benzo[*b*]thiophene H3), 7.98–8.04 ppm (2 H, m, 2×benzo[*b*]thiophene H). MS (LCMS ES+): *m*/*z* (%) 207 (68) [*M*+2H]^2+^, 329 (26) [*M*−piperidine]^+^, 414 (100) [*M*+H]^+^. HRMS (ES+): calcd for C_24_H_36_N_3_O_1_S_1_ [*M*+H]^+^ 414.2574, found 414.2579 (−1.23 ppm).

**4′-(Benzo[*b*]thiophen-2-yl)-1′-benzyl-1,4′-bipiperidine 34**: LiAlH_4_ (0.29 mmol, 2.0 m in THF, 143 μL) was added to a solution of **30** (0.095 mmol, 39 mg) in anhyd THF (3 mL) and the reaction heated at 40 °C for 3 h before the reaction was quenched by the careful addition of aq HCl (10 %, 5 mL). The aqueous phase was then adjusted to pH 10 by the addition of aq NaOH (2 m) and extracted with EtOAc (3×10 mL). The combined EtOAc layers were dried (MgSO_4_), filtered and concentrated in vacuo. The crude product was purified by flash column chromatography (CH_3_OH/CH_2_Cl_2_, 0:100→10:90+1 % NH_4_OH) to give a clear glass (6 mg, 15 %). The reported analysis is for the HCl salt. ^1^H NMR (500 MHz, CD_3_OD): *δ*=1.30–1.37 (1 H, m, CH_2_CH*H*CH_2_), 1.75–1.82 (1 H, m, CH_2_CH*H*CH_2_), 1.95–2.02 (4 H, m, C*H*_2_CH_2_C*H*_2_), 2.77–2.84 (4 H, m, 2×CH_2_), 3.09–3.23 (4 H, m, 2×CH_2_), 3.68–3.73 (2 H, m, 2×C*H*H), 3.79–3.85 (2 H, m, 2×C*H*H), 4.28 (2 H, s, C*H*_2_Ph), 7.48–7.57 (7 H, m, 5×PhH & 2×benzo[*b*]thiophene H), 7.91 (1 H, s, benzo[*b*]thiophene H3), 8.00–8.04 ppm (2 H, m, 2×benzo[*b*]thiophene H). MS (LCMS ES+): *m*/*z* (%) 196 (86) [*M*+2H]^2+^, 306 (100) [*M*−piperidine]^+^, 391 (38) [*M*+H]^+^. HRMS (ES+): calcd for C_25_H_31_N_2_S_1_ [*M*+H]^+^ 391.2202, found 391.2197 (1.50 ppm).

**2-(4′-(Benzo[*b*]thiophen-2-yl)-1,4′-bipiperidinyl-1′-yl)-*N***,***N*****-dimethylethanamine 35**: LiAlH_4_ (0.21 mmol, 2.0 m in THF, 105 μL) was added to a solution of **32** (0.07 mmol, 27 mg) in anhyd THF (0.5 mL) and the reaction heated at 40 °C for 30 min, before the reaction was quenched by the careful addition of water. The aqueous phase was then adjusted to pH 10 by the addition of aq NaOH (2 m) and the aqueous phase extracted with CH_2_Cl_2_ (3×10 mL), the combined CH_2_Cl_2_ layers were dried (MgSO_4_), filtered and concentrated in vacuo. The crude product was purified by flash column chromatography (CH_3_OH/CH_2_Cl_2_, 0:100→10:90+1 % NH_4_OH) to give a white solid (11 mg, 42 %). The reported analysis is for the HCl salt. Note, peaks are broad and poorly defined making assignment of the spectra difficult. ^1^H NMR (500 MHz, CD_3_OD): *δ*=1.31–1.39 (1 H, m, CH*H*), 1.76–1.82 (1 H, m, CH*H*), 1.92–2.03 (4 H, m), 2.74–2.85 (4 H, m), 2.98 (6 H, s, 2×CH_3_), 3.11–3.24 (3 H, m), 3.42–3.80 (7 H, m), 3.83–4.89 (2 H, m), 7.51–7.55 (2 H, m, 2×benzo[*b*]thiophene H), 7.91 (1 H, s, benzo[*b*]thiophene H3), 7.99–8.03 ppm (2 H, m, 2×benzo[*b*]thiophene H). MS (LCMS ES+): *m*/*z* (%) 186 (100) [*M*+2H]^2+^, 287 (24) [*M*−piperidine]^+^, 372 (39) [*M*+H]^+^. HRMS (ES+): calcd for C_22_H_34_N_3_S_1_ [*M*+H]^+^ 372.2468, found 372.2464 (1.11 ppm).

***cis*****- &** ***trans*****-1-(Benzo[*b*]thiophen-2-yl)-4-*tert*-butylcyclohexanol 36**: A solution of *n*BuLi (40 mmol, 1.6 m in hexanes, 25 mL) was added to a solution benzo[*b*]thiophene (40 mmol, 5.37 g) in anhyd THF (100 mL) at −78 °C and stirred for 2 h. The ArLi solution was then added via a cannula to a suspension of CeCl_3_ (40 mmol, prepared by heating 14.9 g CeCl_3_.7H_2_O at 150 °C for 6 h under vacuum) in THF (50 mL) at −78 °C for 30 min. A solution if 4-*tert*-butylcyclohexanone (36 mmol, 5.55 g) in anhyd THF (20 mL) was added to the arylcerium solution and the reaction allowed to warm to 25 °C and stirred for 16 h. The workup was initiated by the addition of saturated aq NH_4_Cl (100 mL), the layers were separated and the aqueous phase extracted with CH_2_Cl_2_ (3×100 mL). The combined organics were dried (MgSO_4_), filtered and concentrated in vacuo. The resultant cream solid was purified by flash column chromatography (EtOAc/Hexane, 0:100→10:90) to give a mixture of *cis* and *trans* isomers as a white solid (7.56 g, 73 %). Note, a small aliquot of the product was further purified to separate the isomers for analytical purposes. Note, the assignment of the isomers as *cis*, or *trans* is made by comparison of the shifts of the *tert*-butyl peaks in the ^1^H NMR spectra as compared to those published for 1-phenyl-4-*tert*-butyl-cyclohexanol.^22^

**For** ***cis*****-36**: *R*_f_=0.34 (EtOAc/hexanes, 1:9). ^1^H NMR (500 MHz, CDCl_3_): *δ*=0.93 (9 H, s, *t*Bu), 1.10–1.16 (1 H, m, CH), 1.51–1.60 (2 H, m, 2×CHC*H*H), 1.72–1.77 (2 H, m, 2×CHC*H*H), 1.86–1.94 (2 H, m, 2×CC*H*H), 2.10–2.15 (2 H, m 2×CC*H*H), 7.19 (1 H, s, benzo[*b*]thiophene H-3), 7.27–7.35 (2 H, m, benzo[*b*]thiophene H5 & H6), 7.71 (1 H, d, *J*=8.0 Hz, benzo[*b*]thiophene H-7), 7.81 ppm (1 H, d, *J*=8.0 Hz, benzo[*b*]thiophene H-4). ^13^C NMR (125 MHz, CDCl_3_): *δ*=22.8 (CH*C*H_2_), 27.6 (CH_3_), 32.5 (*t*Bu C), 40.2 (C*C*H_2_), 47.4 (CH), 72.1 (COH), 117.9 (benzo[*b*]thiophene C3), 122.4 (benzo[*b*]thiophene C4), 123.3 (benzo[*b*]thiophene C7), 123.9 (benzo[*b*]thiophene CH), 124.2 (benzo[*b*]thiophene CH), 139.0 (benzo[*b*]thiophene C), 139.9 (benzo[*b*]thiophene C), 155.9 ppm (benzo[*b*]thiophene C). MS (LCMS ES+): *m*/*z* (%) 271 (100) [*M*−OH]^+^, 599 (12) [2*M*+Na]^+^.

**For** ***trans*****-36**: *R*_f_=0.16 (EtOAc/hexanes, 1:9). ^1^H NMR (500 MHz, CDCl_3_): *δ*=0.81 (9 H, s, *t*Bu), 1.15–1.25 (3 H, m, CH & 2×CHC*H*H), 1.82–1.91 (4 H, m, 2×CHC*H*H & 2×CC*H*H), 2.49–2.53 (2 H, m, 2×CC*H*H), 7.32–7.39 (3 H, m, benzo[*b*]thiophene H-3, H-5 & H-6), 7.76–7.77 (1 H, m, benzo[*b*]thiophene H-7), 7.85 ppm (1 H, d, *J*=7.5 Hz, benzo[*b*]thiophene H-4). ^13^C NMR (125 MHz, CDCl_3_): *δ*=25.0 (CH*C*H_2_), 27.6 (CH_3_), 32.3 (*t*Bu C), 40.0 (C*C*H_2_), 47.6 (CH), 72.7 (COH), 121.0 (benzo[*b*]thiophene C3), 122.4 (benzo[*b*]thiophene C4), 123.6 (benzo[*b*]thiophene C7), 124.2 (benzo[*b*]thiophene CH), 124.4 (benzo[*b*]thiophene CH), 139.5 (benzo[*b*]thiophene C), 139.9 (benzo[*b*]thiophene C), 151.2 ppm (benzo[*b*]thiophene C). MS (LCMS ES+): *m*/*z* (%) 271 (100) [*M*−OH]^+^.

**1-(Benzo[*b*]thiophen-2-yl)-4-*tert*-butylcyclohexanamine 37**: NaN_3_ (20 mmol, 1.30 g) was carefully added to a solution of TCA (10 mmol, 1.63 g) in CHCl_3_ (50 mL) at −20 °C. After stirring for 15 min a solution of *cis*/*trans*-**36** (6.5 mmol, 1.87 g) in CHCl_3_ (200 mL) was added drop-wise and the reaction allowed to warm to 0 °C and stirred for a further 30 min. Workup was initiated by pouring the reaction into water (200 mL) followed by adjusting the aqueous layer to pH 9 (aq NH_4_OH), before the layers were separated and the aqueous phase extracted with CHCl_3_ (3×200 mL), the CHCl_3_ layers were combined, dried (MgSO_4_), filtered and concentrated in vacuo to give a mixture of the cis/trans-azide contaminated with olefin elimination product, which was further reacted without purification. A solution of LiAlH_4_ (20 mmol, 1.0 m in THF, 20 mL) was added to a solution of the crude azide in anhyd Et_2_O (20 mL) in a reflux apparatus and the reaction stirred at 25 °C for 2 h before the reaction was quenched by the addition of water (20 mL) followed by aq NaOH (2 m, 20 mL). Subsequently the biphasic mixture was filtered through Celite, the Celite washed with THF (20 mL), the layers separated and the aqueous phase extracted with Et_2_O (2×40 mL) and the organics combined and concentrated in vacuo. The resultant crude mixture was partitioned between Et_2_O (100 mL) and aq citrate (10 %, 100 mL) and further worked up as described in method A above to give a white solid (84 mg, 2 % over two steps), which was used without further purification. MS (LCMS ES+): *m*/*z* (%) 271 (100) [*M*−NH_2_]^+^.

**1-(1-(Benzo[*b*]thiopehn-2-yl)-4-*tert*-butylcyclohexyl)piperidine 38**: To a suspension of amine **37** (0.3 mmol, 84 mg) and K_2_CO_3_ (1.35 mmol, 186 mg) in anhyd CH_3_CN (10 mL) was added 1,5-dibromopentane (0.66 mmol, 152 mg). The subsequent reaction mixture was heated at reflux for 84 h, filtered and concentrated in vacuo. The resultant crude product was partitioned between H_2_O and Et_2_O (1:2, 75 mL), the layers separated and the aqueous phase extracted with Et_2_O (2×50 mL). The combined Et_2_O layers were then extracted with aq citrate (10 %, 3×100 mL), the combined aqueous layers were then basified to pH 10 (aq NH_4_OH) and subsequently extracted with EtOAc (3×100 mL). The combined EtOAc layers were dried (MgSO_4_), filtered and concentrated in vacuo. The crude product was purified by flash column chromatography (EtOAc/Hexane, 0:100→50:50) to give *cis*- and *trans*-**38**, the latter of which was further purified by trituration of the HCl salt from Et_2_O.

**For** ***cis*****-38**: *R*_f_=0.77 (EtOAc/hexanes, 1:9). The reported analysis is for the free base. Note, 1H NMR analysis was performed at 50 °C due to broad peaks. ^1^H NMR (500 MHz, CDCl_3_): *δ*=0.84 (9 H, s, *t*Bu CH_3_), 1.03–1.09 (1 H, m, CH), 1.23–1.28 (2 H, m, 2×C*H*H), 1.44–1.57 (10 H, m, 2×C*H*H, 3×CH_2_ & 2×CCH*H*), 2.33–2.38 (4 H, m, 2×NCH_2_), 2.47–2.51 (2 H, m, 2×CCH*H*), 6.93 (1 H, s, benzo[*b*]thiophene H3), 7.14–7.23 (2 H, m, benzo[*b*]thiophene H5 & H6), 7.62 (1 H, d, *J*=8.0 Hz, benzo[*b*]thiophene H), 7.69 ppm (1 H, d, *J*=8.0 Hz, benzo[*b*]thiophene H). ^13^C NMR (125 MHz, CDCl_3_): *δ*=21.9 (CH_2_), 25.0 (CH_2_), 27.3 (CH_2_), 27.6 (CH_3_), 32.6 (*t*Bu C), 36.1 (C*C*H_2_), 46.2 (NCH_2_), 47.7 (CH), 58.8 (C), 119.1 (benzo[*b*]thiophene CH), 122.0 (benzo[*b*]thiophene CH), 123.1 (benzo[*b*]thiophene CH), 123.4 (benzo[*b*]thiophene CH), 123.9 (benzo[*b*]thiophene CH), 138.5 (benzo[*b*]thiophene C), 139.6 (benzo[*b*]thiophene C), 149.9 ppm (benzo[*b*]thiophene C). MS (LCMS ES+): *m*/*z* (%) 271 (100) [*M*−piperidine]^+^, 356 (92) [*M*+H]^+^. HRMS (ES+): calcd for C_23_H_34_N_1_S_1_ [*M*+H]^+^ 356.2406, found 356.2405 (0.49 ppm).

**For** ***trans*****-38**: *R*_f_=0.08 (EtOAc/hexanes, 1:9). The reported analysis is for the HCl salt. ^1^H NMR (500 MHz, CD_3_OD): *δ*=0.68 (9 H, s, *t*Bu CH_3_), 1.09–1.24 (4 H, m, 2×CH*H* & CH_2_), 1.62–1.76 (3 H, m, 2×CH*H* & CH), 1.83–1.92 (6 H, m, & 4×CH*H* & 2×CCH*H*), 2.57–2.63 (2 H, m, 2×NCH*H*), 2.86–2.90 (2 H, m, 2×CCH*H*), 3.69–3.74 (2 H, m, 2×NCH*H*), 7.35–7.38 (2 H, m, 2×benzo[*b*]thiophene H), 7.65 (1 H, s, benzo[*b*]thiophene H3), 7.81–7.87 ppm (2 H, m, 2×benzo[*b*]thiophene H). ^13^C NMR (125 MHz, CD_3_OD): *δ*=22.9 (CH_2_), 24.9 (CH_2_), 25.4 (CH_2_), 27.7 (CH_2_), 32.9 (C), 35.0 (C*C*H_2_), 47.8 (CH), 49.4 [under CD_3_OD, identified by DEPT135 & HSQC] (N CH_2_), 71.3 (C), 123.4 (benzo[*b*]thiophene CH), 125.8 (benzo[*b*]thiophene CH), 126.3 (benzo[*b*]thiophene CH), 127.2 (benzo[*b*]thiophene CH), 130.0 (benzo[*b*]thiophene C3), 140.7 (benzo[*b*]thiophene C), 141.6 ppm (benzo[*b*]thiophene C) [Note, one quaternary carbon is missing, or two carbons have identical shifts]. MS (LCMS ES+): *m*/*z* (%) 271 (100) [*M*−piperidine]^+^, 356 (92) [*M*+H]^+^. HRMS (ES+): calcd for C_23_H_34_N_1_S_1_ [*M*+H]^+^ 356.2406, found 356.2408 (−0.32 ppm).
